# Reverse transcriptases prime DNA synthesis

**DOI:** 10.1093/nar/gkad478

**Published:** 2023-06-06

**Authors:** Matej Zabrady, Katerina Zabrady, Arthur W H Li, Aidan J Doherty

**Affiliations:** Genome Damage and Stability Centre, School of Life Sciences, University of Sussex, Brighton BN1 9RQ, UK; Genome Damage and Stability Centre, School of Life Sciences, University of Sussex, Brighton BN1 9RQ, UK; Genome Damage and Stability Centre, School of Life Sciences, University of Sussex, Brighton BN1 9RQ, UK; Genome Damage and Stability Centre, School of Life Sciences, University of Sussex, Brighton BN1 9RQ, UK

## Abstract

The discovery of reverse transcriptases (RTs) challenged the central dogma by establishing that genetic information can also flow from RNA to DNA. Although they act as DNA polymerases, RTs are distantly related to replicases that also possess *de novo* primase activity. Here we identify that CRISPR associated RTs (CARTs) directly prime DNA synthesis on both RNA and DNA. We demonstrate that RT-dependent priming is utilized by some CRISPR-Cas complexes to synthesise new spacers and integrate these into CRISPR arrays. Expanding our analyses, we show that primer synthesis activity is conserved in representatives of other major RT classes, including group II intron RT, telomerase and retroviruses. Together, these findings establish a conserved innate ability of RTs to catalyse *de novo* DNA primer synthesis, independently of accessory domains or alternative priming mechanisms, which likely plays important roles in a wide variety of biological pathways.

## Graphical Abstract

**Graphical Abstract:**
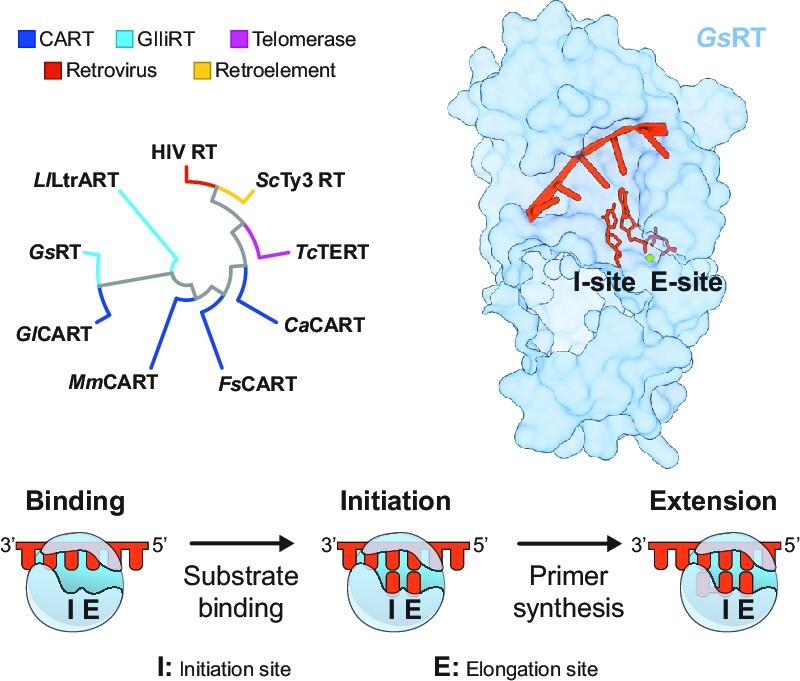

## INTRODUCTION

The replication of genetic information is essential for the propagation of all organisms and this role is performed by replicative polymerases, which efficiently duplicate strands of nucleic acids and give rise to two copies of the genome. Polymerase activities are also required for other genome maintenance pathways, including DNA repair and damage tolerance. Replicative polymerases can be divided into two broad classes, depending on their template preference, namely DNA-dependent and RNA-dependent polymerases. While cellular organisms possess genomes composed of DNA, many viruses encode their genetic information within RNA. Over half a century ago, Baltimore and Temin independently discovered that eukaryotic RNA retroviruses encode RNA-dependent DNA polymerases called reverse transcriptases (RTs) involved in viral genome replication (1,2).

Reverse transcriptases represent a key evolutionary stage in nucleic acid replication as organisms transitioned away from RNA to DNA centric genomes (3,4). RTs belong to a major lineage of replicases, which includes viral RNA-dependent RNA polymerases (RdRPs) and Primase-Polymerases (Prim-Pols). In common with other polymerases, the catalytic ‘palm’ subdomains evolved from an ancestral RNA Recognition Motif like (RRM-like) fold, containing advantageously placed active site residues that nucleate metal-dependent catalysis (5–13). While most RdRPs and Prim-Pols retained the ability to both prime and extend on RNA and /or DNA templates, presumably inherited from ancestral progenitors, RTs appeared to be an exception having apparently lost this innate primase activity.

It has been recognised that RTs also play diverse roles in cells, including retrotransposition, telomere maintenance and Clustered Regularly Interspaced Short Palindromic Repeats (CRISPR) adaptation. The general mechanisms of DNA synthesis by RTs have been extensively studied and although a number of unorthodox RT-specific priming mechanisms have been reported (14), including endonuclease-produced 3’ termini and tRNAs that act as surrogate primers, the putative priming mechanism(s) of other RTs remains to be elucidated. Given the near universal usage of *de novo* primer synthesis to initiate DNA replication, it is conspicuous that an equivalent mechanism has not been identified within RT-dependent replicative complexes.

A highly divergent group of RTs, predominantly related to group II intron RTs (GIIiRT), are genetically associated with a subset of Type III CRISPR-Cas operons and reportedly play roles in spacer acquisition from RNA (15–20). In this study, we investigated the activities of CRISPR-associated reverse transcriptases (CARTs) and discovered a previously unidentified DNA priming mechanism. We show how this primer synthesis activity is utilised to facilitate the integration of spacers, originating from RNA, into CRISPR arrays. We also expand our study to other major phylogenetic groups of RTs and establish the conservation of *de novo* primer synthesis across the RT superfamily.

## MATERIALS AND METHODS

### Bioinformatics

Sequences of RTs, previously identified to associate with CRISPR, were extracted from their respective original publications (18,21,22). Sequences were aligned using MUSCLE (23) and phylogenetic tree was build using FastTree 2 (24). The RT domain type was inferred using myRT tool (25).

### Cloning, expression and purification of recombinant proteins

Table of plasmids used in this study is provided in Supplementary Table S1. The sequence of all cloned genes was codon optimised for expression in *Escherichia coli* Bl21 using IDT’s Codon Optimization Tool. Table of recombinant protein expression and purification conditions is provided in Supplementary Table S2. In general, for protein expression BL21 Star (DE3) *E. coli* strain (Invitrogen) was used, using TB medium (Molecular Dimensions, MD12-104-01) and induced by 1 mM isopropyl β-d-1-thiogalactopyranoside (IPTG) either as overnight expression at 16°C or 3-h expression at 37°C. The cells were disrupted by sonication and centrifuged at 50 000 g for 30 min, proteins were purified by indicated purification methods found in Supplementary Table S2, and flash-frozen in liquid nitrogen as 50% glycerol stocks. Two μg of purified proteins used in this study were run on SDS-PAGE and Coomassie stained (Supplementary Figure S1).

### Polymerase assay

All standard polymerase reactions were assayed in a 20 μl volume, containing 10 mM buffer, 10 mM divalent metal cation, 0.2 mM dNTP mix, 50 nM FAM-labelled primer strand annealed with a template strand (oPK404-407, Supplementary Table S3) and 50 nM protein. Detailed reaction conditions for each assay are displayed in Supplementary Table S4. After indicated time, the reactions were stopped with an equal volume of CTAB buffer (200 μM cetyltrimethylammonium bromide (CTAB), 30 mM (NH_4_)_2_SO_4_, 25 mM ethylenediaminetetraacetic acid (EDTA)) and centrifuged at 16 000 g to precipitate nucleic acids. The supernatant was aspirated and the pellet dissolved in sample loading buffer (92.5% formamide, 25 mM EDTA, 0.5% Ficoll 400), boiled at 95°C for 3 min, loaded onto 10% TBE–urea PAGE gel (10% acrylamide/bis-acrylamide, 19:1, 8 M urea and 1× Tris/borate/EDTA (TBE)) and resolved for 90 min at 25 W. Gel were imaged on a FLA 5100 scanner (FujiFilm) and final image was adjusted in FIJI (26) using the linear range.

### Fluorescence polarization assay

Equilibrium binding reactions were assayed in a 50 μl volume, containing 10 mM Tris pH 7.5, 10 mM MnCl_2_, 0.05% Tween-20, 50 nM FAM-labelled primer strand annealed with a template strand (oPK404-407, Supplementary Table S3) and increasing protein concentration up to a value, which started to influence parallel fluorescence. Fluorescent polarization was measured by CLARIOstar (BMG Labtech) with filter settings (482–16/LP 504/530–40) and results normalized by subtracting background fluorescent polarization. Data was fitted with Hill–Langmuir equation and plotted using Python.

### Gel-based primase assay

All standard gel-based primase reactions were assayed in a 20 μl volume, containing 10 mM buffer, 10 mM divalent metal cation, 1:10 ratio of labelled and unlabelled nucleotide (Supplementary Table S3), 1 μM template (Supplementary Table S3) and 1 μM protein. Detailed reaction conditions are shown in Supplementary Table S5. After indicated time, the reactions were processed the same as polymerase assay samples, loaded onto 20% TBE–urea PAGE (20% acrylamide/bis-acrylamide, 19:1, 8 M urea and 1× TBE) gel and resolved for 120 min at 25 W. Gels were imaged on a FLA 5100 scanner (FujiFilm) and final image was adjusted in FIJI (26) using the linear range. For quantification, the signal of each sample in individual lanes without signal of the fluorescently labelled mononucleotide signal was measured using FUJI (26) and the background signal of the no protein control was subtracted from each protein sample lane from the same gel. The signal of priming products of HsPrimPol was used as a standard (100%) for other samples from the same gel. Python was used to calculate mean, SD and create the bar graph.

### Intercalating fluorescent dye-based primase assay

Basic principle of this assay exploits the use of 3’ end phosphorylated templates and GelRed (Biotium) intercalating fluorescent dye to detect dsDNA formation. All standard reactions were assayed in 50 μl volume, containing reaction components indicated in Supplementary Table S6. Fluorescence intensity was measured by CLARIOstar (BMG Labtech) with filter settings (545–20/600–20) over the course of 15–30 min. The time course was plotted as an average of three independent reactions with standard error highlighted (CI 95) in python. The first-order reaction rates were compared after linear regression fit of the linear portion of curves and statistical analysis using python. The data for Michaelis constant (*K*_m_) of dGTP was fitted as slopes of the linear regression fit of the reaction rates with Hill–Langmuir equation and plotted using Python. Statistical analysis was performed using statannotations Python package with two-tailed independent t-test.

### Pyrophosphate luminescence-based primase assay

Basic principle of this coupled assay combines the release of pyrophosphate (PPi) upon phosphodiester-bond formation with the luminescent detection of PPi by PPiLight kit (Lonza). All standard reactions were assayed at room temperature in 50 μl volume, containing 10 mM Tris pH 7.5, 1 mM MnCl_2_, 1 μM oMZ13, 10 nM protein and varying dGTP concentrations. The data for Michaelis–Menten constant of dGTP was fitted as slopes of the linear regression fit of the reaction rates using the Hill–Langmuir equation and plotted using Python.

### DNA strand-displacement assay

Strand displacement assays involving gapped DNA substrates were performed as previously reported (22,27). In brief, substrates were formed by annealing oligonucleotides oNB1 and oNB2 (22,27) with oNB8–11 (Supplementary Table S3). 50 nM substrate was mixed with 100 μM dNTPs and 50 nM *Ca*CART or 100 nM *Mm*CART. The reactions were incubated at 37°C for 30 min, reaction was processed as per polymerase assay samples, loaded onto 10% TBE–urea PAGE gel and resolved for 90 min at 25 W. Gels were imaged on a FLA 5100 scanner (FujiFilm) and the images were adjusted in FIJI (26) using the linear range.

### *In vitro* prespacer integration assay

10 μM *Mm*Cas6-RT-Cas1 with 20 μM *Mm*Cas2 were premixed together in Protein dilution buffer (50 mM HEPES; pH7.5, 250 mM NaCl, 10% glycerol and 0.5 mM TCEP). 1 μl of *Mm*Cas6-CART-Cas1 + *Mm*Cas2 premix was mixed with 5 μl of H_2_O and 1 μl of 10x buffer A (500 mM Bis–Tris propane; pH7, 100 mM MnCl_2_ or MgCl_2_ and 100 mM TCEP), 1 μl of 2 μM prespacer ± 1 μl of 1 mM dNTPs and 1 μl of 250 nM CRISPR array A, respectively, and incubated for 1 hour at 37°C. The reactions were stop by addition of 10 μl of Stop buffer (60% formamide, 6 M urea, 5 mM EDTA, 0.025% SDS) boiled at 95°C for 3 min and resolve on 10% urea-PAGE (19:1 Acrylamide/Bis-acrylamide, 8 M urea) in 1× TBE for 90 min at 25 W. The gel was imaged on a FLA 5100 (FujiFilm) and images adjusted in FIJI (26) in the linear range. Prespacers: ssDNA^FW^—oKZ464, ssDNA^REV^—oKZ508, ssRNA^FW^—oKZ465, ssDNA^FW^ + ssDNA^REV^—oKZ464 + oKZ508, ssRNA^FW^ + ssDNA^REV^—oKZ465 + oKZ508.

### *In vitro* primed prespacer integration assay

10 μM MmCas6-CART-Cas1 and 20 μM MmCas2 were premixed together in Protein dilution buffer and 20 μl of the premixed protein was added to the 200 μl reaction (final volume) containing 50 mM Bis-Tris Propane; pH7, 10 mM MnCl_2_, 10 mM TCEP, 10 μM γ-phosphate FAM-labelled GTP (FAM-γGTP), 100 μM dTTP, dGTP and dCTP and 1 μM short ssRNA template (oKZ535). The reaction was incubated at 37°C for 15 min before addition of CRISPR array B substrate (25 nM final). The reaction was incubated at 37°C for 60 min before stopping by addition of 4 μl of Proteinase K (0.8 U/ul—NEB) and incubated for another 30 min at 37°C. 25 μl of the reaction (INPUT fraction) was mixed with 25 μl of CTAB buffer and spun for 10 min at 16000 g at room temperature. The pellet was resuspended in 20 μl of sample loading buffer (92.5% formamide, 25 mM EDTA, 0.5% Ficoll 400) before loading on urea-PAGE.

8.75 μl of 200 mM PMSF was added into the remaining 175 μl of the reaction to inactivate proteinase K. 2 μl of the reaction was mixed with 8 μl of H_2_O, boiled for 10 min and 1 μl was used in 10 μl PCR reaction. 70 μl of magnetic streptavidin beads (Merck, Roche—11641786001) and 200 μl of protein dilution buffer containing 25 mM EDTA was added to the remaining reaction followed by incubation for 15 min at room temperature. The magnetic beads were washed 3 times with 500 μl of Protein dilution buffer and resuspended with 100 μl of water and 200 μl of DNA Cleanup Binding Buffer (Monarch® PCR & DNA Cleanup Kit, NEB) and incubated for 5 min at room temperature before addition of 600 μl of 100% Et-OH. The sample was loaded to the Monarch® DNA cleanup columns following NEB oligonucleotide cleanup protocol. The samples were eluted with 20 μl of sample loading buffer—BOUND fraction. All samples (input and bound fractions) were boiled for 3 min and resolve on 10% urea–PAGE (19:1 acrylamide/bis-acrylamide, 8 M urea) in 1× TBE for 75 min at 25 W. Gel was imaged on a FLA 5100 (FujiFilm) and images adjusted in FIJI (26) in the linear range.

### PCR, cloning and sequencing of *in vitro* primed prespacer integration assay products

Phusion polymerase (NEB) in combination with GC buffer was used to amplify Cas1–Cas2 integrated products in CRISPR array using primers oKZ536 and oKZ537 which include adaptors for NEBuilder HiFi DNA assembly cloning into *Hind*III digested pUC19. Primer oKZ537 binds to the repeat-spacer end of the CRISPR array and oKZ537 binds only if the integrated DNA strand is a priming product of RT using RNA template oKZ535. PCR conditions: *T*_a_—61°C, extension time—10 s, 20 cycles. The PCR product was resolved on 2% agarose gel in 1× TAE containing ethidium bromide. Gel images were adjusted in FIJI (26) using the linear range.

After gel extraction, the PCR product was cloned into *Hind*III digested pUC19 using NEBuilder HiFi DNA assembly cloning kit (NEB). The cloning products were transformed into *E. coli* and the plasmid from 22 single colonies was isolated and send form Sanger sequencing. The sequencing results were analysed using QIAGEN CLC Main Workbench. For the alignment, the sequence of pUC19 was omitted. For simplicity 5 sequenced samples were omitted in Figure 4e, however, all samples are shown in the Supplementary Figure S4.

### Preparation of DNA / RNA substrates

Sequences and modifications of the synthetic oligonucleotides used are shown in Supplementary Table S2. Overhangs and gapped substrates for polymerase and strand-displacement assays were prepared by mixing equimolar amounts of the corresponding oligonucleotides in buffer containing 10 mM Tris pH 7.5 and 50 mM NaCl, heating at 95 °C for 3 min and cooling slowly to room temperature.

The CRISPR array A was prepared by PCR amplification using pKZ223 plasmid as a template and primers oKZ500 and oKZ501, Phusion polymerase and GC buffer, *T*_a_—58°C, extension time—15 s, 40 cycles in 200 μl. The products were resolved on 10% native PAGE (37.5:1 acrylamide/bis-acrylamide) in 1× TBE for 1 h at 120 V, the band containing labelled PCR product was cut out and eluted from the crushed gel into water by diffusion for 1 h at room temperature. The DNA was precipitated with ethanol and resuspended in water.

The CRISPR array B was prepared by PCR amplification using pKZ223 plasmid as a template and primers oKZ515 and oKZ503, Phusion polymerase and GC buffer, *T*_a_—58°C, extension time—15 s, 40 cycles in 400 μl. The PCR product was precipitated with ethanol and resuspended in 25 μl of H_2_O and then loaded on Dye Ex 2.0 (Qiagen) to remove free nucleotides. Eluted DNA was 3’ labelled by Cy5-ddCTP (Jena Bioscience, NU-850-CY5) using Terminal Deoxynucleotidyl Transferase (TdT) (Thermo Fisher Scientific, EP0161) in 80 μl reaction containing DNA, 0.1 mM Cy5-ddCTP, 15 μl of 5× reaction buffer and 140 U of TdT which was incubated for 2 h at 37°C. The products were resolved on 10% native PAGE (37.5:1 Acrylamide/Bis-acrylamide) in 1× TBE for 75 min at 120 V, the band containing labelled DNA fragment was cut out and eluted from the crushed gel into water by diffusion for 1 h at room temperature. The DNA was ethanol precipitated and resuspended in 200 μl of water.

### Crystallisation, data collection and structure determination

Crystal screening experiments were set up with 500 mM *Ca*CART-CAPP_RT (aa1-204) domain and matrix screens (Molecular Dimensions, Hampton Research) using sitting-drop, vapour-diffusion method, with equal volumes of protein solution and reservoir buffer. Crystal was grown in 20% PEG 3350 and 0.2 M sodium tartrate dibasic dihydrate, and cryoprotected in the mother liquor with 25% PEG 400. Diffraction data were collected at beamline I24 of Diamond Light Source (Didcot, UK).

The diffraction data were processed with xia2 (28) and DIALS (29). Molecular replacement with Phaser (30) was performed using 6ar1 (31) as a template, followed by alternate rounds of manual and automatic refinement of the model using Coot (32) and phenix.refine (33). Molecular graphics were generated with PyMOL (Schrödinger, LLC) (34) and ChimeraX (UCSF) (35). Structural alignment was performed using the Dali server (36).

## RESULTS

### DNA polymerase activities of *Ca*CART-CAPP

**Figure 1.**
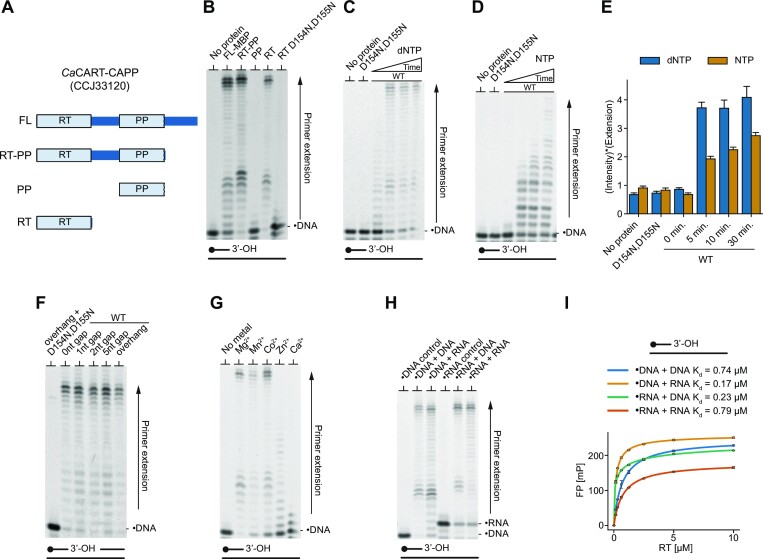
DNA polymerase activities of *Ca*CART-CAPP. (**A**) Graphical representation of protein domain constructs derived from *Ca*CART-CAPP (CCJ33120). Reverse transcriptase domain, RT; Prim-Pol domain, PP; FL, full length. (**B**) *Ca*CART-CAPP polymerase activity is attributable to the RT domain. 50 nM 5’ end FAM-labelled DNA primer (oPK405) annealed with DNA template (oPK404), was extended with 50 nM protein, in presence of Mg^2+^ and dNTPs for 30 min. FL-MBP represents full-length protein with C-terminal MBP fusion. (**C–E**) The *Ca*CART-CAPP RT domain shows efficient primer extension. Time course: 0, 5, 10 and 30 min. No protein control and RT D154N, D155N mutant were incubated for 30 min. FAM-labelled DNA primer (oPK405) annealed with 50 nM DNA template (oPK404) was incubated with 50 nM RT domain in presence of Mn^2+^ and dNTPs (C), or NTPs (D) and the signal of extended primers from panels C and D was quantified by plotting the signal intensity, weighted by an extension factor - the distance of the signal from primer position (E). **(F)** Strand-displacement synthesis activity of the RT domain of *Ca*CART-CAPP. 50 nM protein was incubated with 50 nM DNA substrates oNB1 + oNB2 + oNB8-11 in presence of dNTPs and Mn^2+^. DNA primer: oNB2, DNA template: oNB1, downstream strand (oNB8-11). (**G**) Mg^2+^, Mn^2+^ and Co^2+^ promote efficient DNA extension by RT domain of *Ca*CART-CAPP and with dNTPs (oPK404 + oPK405). (**H**) Primer extension activity of the RT domain of *Ca*CART-CAPP with combinations of DNA/RNA primers annealed to DNA/RNA templates in presence of dNTPs and Mn^2+^. Control is without a protein. Products of all polymerase reactions are resolved on 10% TBE-Urea-PAGE gel (panels B–H). (**I**) The *Ca*CART-CAPP RT domain is indiscriminate in its binding to DNA or RNA substrates. The *K*_d_ recorded by fluorescence polarization assays was similar for every combination of DNA / RNA primer (oPK405 / oPK407) and template (oPK404/oPK406). Dot symbol, FAM-labelled primer (•DNA, •RNA); WT, *Ca*CART-CAPP RT wild type; D154N, D155N catalytic mutant of the *Ca*CART-CAPP RT.

A subset of CARTs are fused with CRISPR-associated Prim-Pols (CAPPs). CAPPs possess both primer synthesis and extension activities, implicated in spacer acquisition in Type III CRISPR-Cas systems (22). To elucidate the role(s) of CARTs in CRISPR-Cas spacer acquisition, we first characterised the synthesis activities associated with the RT domain of *Caloramator australicus Ca*CART-CAPP (CCJ33120). This consists of an N-terminal RT domain fused to a Prim-Pol domain (PP) and C-terminal domain (CTD). We purified full-length *Ca*CART-CAPP (FL; aa1-713) and its derivatives including: RT-PP domains lacking the CTD (RT-PP; aa 1–521); PP domain (PP; aa 322–521); RT domain composed of the fingers-palm subdomains (RT; aa 1–204); RT domain catalytic mutant (RT D154, D155N; aa 1–204), and examined their primer extension activities (Figure 1A). FL possessed DNA-dependent DNA polymerase activity (Figure 1B), comparable to RT-PP, but no apparent activity was evident for PP domain alone. However, RT domain alone exhibited strong extension activity, lacking in a catalytic mutant (D154N, D155N), confirming that the polymerase activity is solely attributable to the RT domain. The primer extension activity of the RT domain is supported by the presence of dNTPs (Figure 1C) and is significantly weaker with NTPs (Figure 1D), quantified in Figure 1E. The RT domain exhibited robust strand-displacement synthesis activity on substrates with different gap sizes (nick to 5 nt) (Figure 1F) and was able to utilize different divalent metal cations (Mg^2+^, Mn^2+^ and Co^2+^) for primer extension activity (Figure 1G). The substrate specificity of the polymerase activity of RT domain was next tested on different combinations of DNA / RNA templates annealed with labelled primers and shown to be equally proficient in primer extension using dNTPs on every nucleic acid substrate combination (Figure 1H). Similar results were obtained by measuring binding affinities for different DNA or RNA substrates using fluorescence polarisation (Figure 1I), suggesting a common binding mechanism for both types of nucleic acids.

### DNA primase activities of *Ca*CART-CAPP

**Figure 2.**
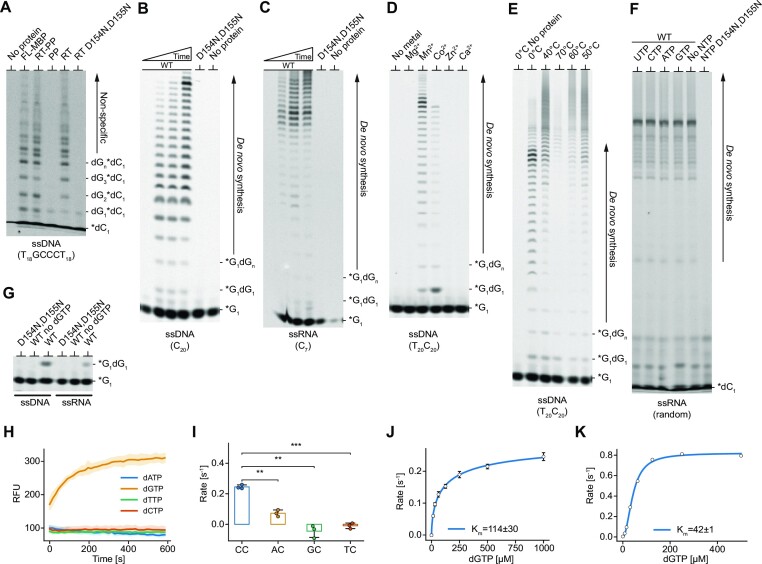
DNA primase activities of *Ca*CART-CAPP. (**A**) *Ca*CART-CAPP primase activity is attributed to the RT domain. The incorporation of dGTP and FAM-labelled dCTP in the newly synthesised DNA primer, complementary to 1 μM DNA template containing 5’-T_18_GCCCT_18_-3’ sequence (oKZ364) by 1 μM protein in presence of Mn^2+^. FL-MBP represents full-length protein with C-terminal MBP fusion. (B, C) *Ca*CART-CAPP RT domain is an efficient DNA and RNA-dependent primase. Time course of the primase activity of RT domain with 1 μM DNA (oMZ13) (panel **B**) or RNA (oMZ17) (panel **C**), 1 μM RT domain of *Ca*CART-CAPP, γ-phosphate FAM-labelled GTP, dGTP and Mn^2+^. Time course: 0, 2, 5 and 30 min. No protein control and RT D154N, D155N mutant were incubated for 30 min. (**D**) Mn^2+^ and Co^2+^ promote DNA primer synthesis by *Ca*CART-CAPP RT domain. 1 μM RT domain, DNA template (oKZ148), dGTP, γ-phosphate FAM-labelled GTP and divalent cation. (**E**) The optimal temperature range for the primase activity of *Ca*CART-CAPP RT domain. Prominent shift in size of the synthesized products with increasing temperature (above 0°C) indicates non-specific extension beyond the template length. Primase assays consists of RT domain, DNA template (oKZ148), dGTP, γ-phosphate FAM-labelled GTP and Mn^2+^. (**F**) *De novo* DNA synthesis by *Ca*CART-CAPP RT domain is not stimulated by presence of NTPs. A random RNA sequence (oKZ151) was used as non-discriminatory template in the reaction together with dNTPs, NTPs, FAM-labelled dCTP and Mn^2+^. (**G**) Dinucleotide synthesis of *Ca*CART-CAPP RT domain. Using 1 μM substrate (DNA, oKZ153; RNA, oMZ11), 1 μM protein, γ- phosphate FAM-labelled GTP and dGTP. Products of all primase reactions are resolved on 20% TBE–urea–PAGE gel (panels A–G). (**H**) The primase activity of *Ca*CART-CAPP RT domain is proficient only with dGTP. Time courses of dsDNA synthesis on homopolymeric substrates (oKZ147, oKZ148) with respective complementary nucleotides (oKZ147 with dTTP or dCTP; oKZ148 with dATP or dGTP), are initiated with 100 nM protein in presence of Mn^2+^ and measured using a dsDNA intercalating fluorescent dye-based assay. (**I**) A minimum of two cytosines on templates act as a strong initiation site for the *de novo* primer synthesis. Initial reaction rates with *Ca*CART-CAPP RT domain on 1 μM DNA substrates containing different initiation sequences (CC, oKZ153; GC, oKZ155; TC, oKZ294; AC, oMZ26—the first initiation cytosine is underlined), initiated with 25 nM protein in presence of Mn^2+^, 500 μM dNTPs complementary to the initiation site sequence and 100 μM dNTP for the subsequent strand extension. DNA synthesis was measured using a dsDNA intercalating fluorescent dye-based assay and statistical analysis with two-tailed independent t-test. **, *P*-value < 0.01; ***, *P*-value < 0.005. (**J, K**) Initial reaction rates of 25 nM *Ca*CART-CAPP RT domain with increasing dGTP and 1 μM homopolymeric cytosine DNA substrate (oMZ13), fitted to Hill-Langmuir equation and with K_m_ calculated. Measured using a dsDNA intercalating fluorescent dye-based assay (panel J) and a pyrophosphate luminescence-based primase assay (panel K). *dC, free or incorporated FAM-labelled dCTP (panels A, F); *G, γ-phosphate FAM-labelled GTP (panels B–E, G); Template and product sequences: 5’ → 3’, WT, *Ca*CART-CAPP RT wild type; D154N, D155N catalytic mutant of *Ca*CART-CAPP RT.

Given the PP domains of CAPPs also possess *de novo* primer synthesis activities, implicated in CRISPR-Cas adaptation processes (22), we next analysed such activities for *Ca*CART-CAPP (FL) and observed that it also exhibited a robust primase activity. Although the RT-PP fragment was primase proficient, PP domain alone was unexpectedly not, correlating with the lack of polymerase activity. In contrast and unexpectedly, the RT domain alone possessed the striking capacity to perform DNA primer synthesis, comparable with the level of activity of FL and RT-PP. Mutation of catalytic residues (D154N, D155N) of the RT domain abolished this activity (Figure 2A). *De novo* primer synthesis can be initiated on both DNA and RNA templates (Figure 2B, C) and is only supported by addition of manganese or cobalt (Figure 2D), at temperatures up to 70°C (Figure 2E). We previously showed that PP domains of CAPPs require a ribonucleotide, with a strong preference for purines, to initiate primer synthesis (22). To determine if this is also the case for CARTs, we characterised the substrate requirements for the primase activity of the RT domain of *Ca*CART-CAPP. The initiation of primer synthesis by RT domain is not directly dependent on NTPs (Figure 2F), but NTPs can be incorporated in the first position and always followed by dNTPs, enabling us to specifically detect primer synthesis products using γ-phosphate FAM-labelled GTP in gel-based assays (Figure 2G). Analysing different DNA template sequences for their ability to promote primer synthesis revealed a clear preference for the initiation of primer synthesis at cytosines, incorporating guanosines to begin the synthesis of a new DNA strand (Figure 2H). A minimum of two cytosines in the template sequence was sufficient to act as a potent initiation site for primer synthesis (Figure 2I). The affinity for dGTP on a homo-polymeric DNA template (C_20_) was approximated by measuring its *K*_m_ with two independent methods, first detecting the dsDNA formation with a DNA intercalating dye (Figure 2J). Second, detecting the release of pyrophosphate upon the phosphodiester bond formation (Figure 2K) that also identified a strong co-operativity of dGTP (Hill coefficient ≥ 2), as expected for dinucleotide formation. In conclusion, RT domain of *Ca*CART-CAPP is a *bona fide* DNA primase and polymerase, that is active on both DNA and RNA templates, initiates *de novo* synthesis with NTPs or dNTPs, and has a strong preference for priming on templates containing a CC sequence.

### Conservation of catalytic activities in CART proteins

**Figure 3.**
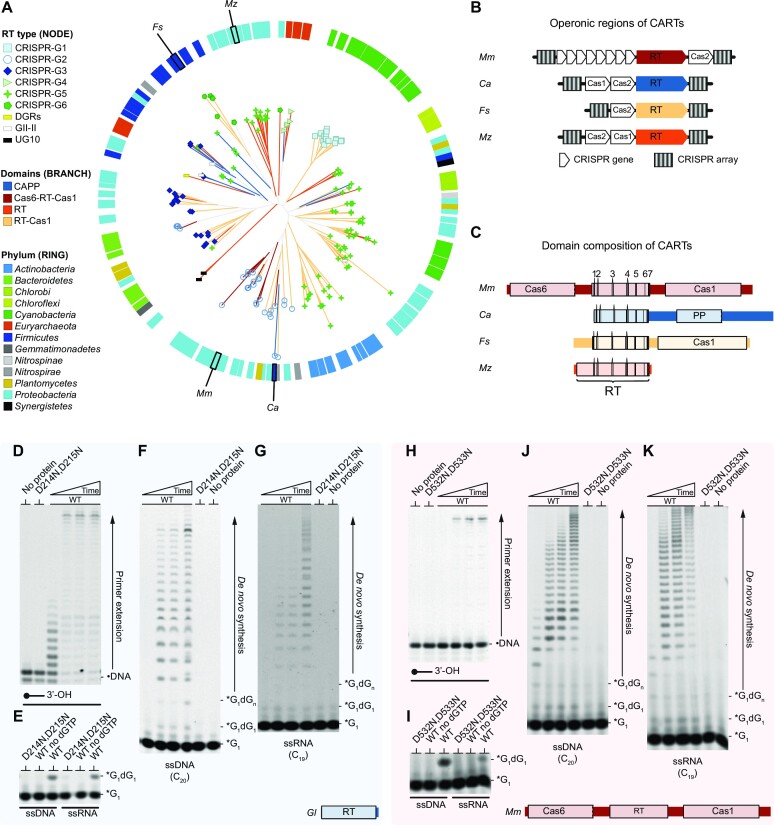
Conservation of the catalytic activities in CARTs. (**A**) Unrooted phylogenetic tree of known CARTs, extracted from previous publications (see Methods). Annotated nodes are based on the RT domain type (myRT), branch colours are based on the presence of other protein domains and outer ring colours are based on the taxonomic unit (phylum) of host organisms. (**B**) Examples of operons for each CART subclass. Based on the presence of other protein domains: Cas6-CART-Cas1 (*Marinomonas mediterranea, Mm*); CART-CAPP (*Caloramator australicus, Ca*); CART-Cas1 (*Fusicatenibacter saccharivorans, Fs*); CART (*Maridesulfovibrio zosterae, Mz*). (**C**) Graphical representation of domain composition of CART subclasses. Highlighted are conserved motifs (1–7) of the reverse transcriptase superfamily. CART, CRISPR associated RT; CAPP, CRISPR Associated Prim-Pol; FL, full length; RT, reverse transcriptase; PP, Prim-Pol. (**D**) The polymerase activity of *Gl*CART-CAPP RT. Time course of the polymerase activity of 50 nM RT domain in presence of 5’ end FAM-labelled DNA primer (oPK405) annealed with DNA template (oPK404), Mn^2+^ and dNTPs. (**E**) Dinucleotide synthesis of *Gl*CART-CAPP RT domain. Reaction contains 1 μM substrate (DNA, oKZ153; RNA, oMZ11), 1 μM protein, γ-phosphate FAM-labelled GTP and dGTP. (**F, G**) DNA and RNA-dependent DNA primase activity of *Gl*CART-CAPP RT domain. Time course with 1 μM DNA (oMZ13) (panel F) or RNA (oMZ27) (panel G), 1 μM protein, γ-phosphate FAM-labelled GTP, dGTP and Mn^2+^. (**H**) The polymerase activity of *Mm*Cas6-CART-Cas1. Time course experiment with 200 nM *Mm*Cas6-CART-Cas1 full-length protein, 50 nM DNA substrate (oPK404 + oPK405), Mg^2+^ and dNTPs. (**l**) Dinucleotide synthesis of *Mm*Cas6-CART-Cas1. Reaction contained 1 μM substrate (DNA, oKZ153; RNA, oMZ11), 1 μM protein, γ-phosphate FAM-labelled GTP and dGTP. (**J**, **K**) DNA and RNA-dependent DNA primase activity of *Mm*Cas6-CART-Cas1. Time course with 1 μM protein, 1 μM DNA (oMZ13) (panel J) or 1 μM RNA (oMZ27) (panel K), γ-phosphate FAM-labelled GTP, dGTP and Mn^2+^. Products of polymerase and primase reactions were resolved on 20% TBE-Urea- PAGE gels (panels D–K). WT, wild type; *G, γ-phosphate FAM-labelled GTP; •DNA, FAM-labelled DNA primer. Time course—polymerase assay (panels D, H): 0, 5, 10 and 30 min.; Time course—primase assay (Panels E–G and I–K): 0, 2, 10 and 30 min; No protein control, GlCART-CAPP RT catalytic mutant D214N, D215N and *Mm*Cas6-CART-Cas1 mutant of the RT domain D532N, D533N were incubated for 30 min.

Known CARTs can be classified, based on their protein domain architecture, into stand-alone (CART), Cas1 fused (CART-Cas1), Cas6 and Cas1 fused (Cas6-CART-Cas1), and CAPP fusions (CART-CAPP) (Figure 3A). CARTs are genetically located near the major CRISPR integrase complex (Cas1 and Cas2) (Figure 3B). Despite the significant divergence of CARTs primary sequences and domain architectures, the characteristic motifs of reverse transcriptase protein superfamily are well conserved, indicating that a common catalytic mechanism is preserved (Figure 3C). To establish if the newly discovered primase activities are conserved across the CART family, we compared the activities of *Ca*CART-CAPP with *Geobacillus lituanicus Gl*CART-CAPP RT domain (aa 1–271) and full-length *Marinomonas mediterranea Mm*Cas6-CART-Cas1. Both *Gl*CART-CAPP RT domain and full length *Mm*Cas6-CART-Cas1 exhibited similar activity profiles as *Ca*CART-CAPP RT domain, including DNA-dependent DNA polymerization (Figure 3D, H), dinucleotide synthesis (Figure 3E, I), *de novo* DNA-dependent DNA synthesis (Figures 2F, 3J), and *de novo* RNA-dependent DNA synthesis (Figure 3G, K). The catalytic mutants of RT domains (*Gl*CART-CAPP RT domain: D214N, D215N and *Mm*Cas6-CART-Cas1: D532N, D533N) were inactive, similar to the equivalent RT mutant of *Gl*CART-CAPP (Supplementary Figure S2A), showing another example of an inactive PP domain in CART-CAPP fusions. Thus, we hypothesize the inactive PP domain is likely a common feature of these multidomain proteins, and the RT domain has replaced its activities. Together, these results establish a prototypical activity profile of CARTs, which utilize a broad range of substrates for the initiation of *de novo* DNA synthesis, and its extension, to potentially facilitate their putative role(s) in CRISPR adaptation.

### Primase activities of CART facilitates integration into CRISPR arrays

To determine the biological relevance of the primase activities of the RT domains, we investigated the adaptation stage of type III CRISPR complexes involving CARTs, as a functional model for understanding their biological role(s). CRISPR-Cas operons containing CARTs can integrate new spacers originating from RNA sources into CRISPR arrays and mutation of the CART domain active site abolishes this activity *in vivo* (15–20). To explain these *in vivo* observations at the molecular level and understand the specific role(s) of CARTs primase activity in the naïve CRISPR adaptation - spacer acquisition step, we conducted *in vitro* spacer integration assays with purified *Marinomonas mediterranea* (*Mm*) integrase complex composed of *Mm*Cas6-CART-Cas1 and *Mm*Cas2. To optimize reaction conditions for these assays, we first analysed the enzymatic properties of *Mm*Cas6-CART-Cas1. This protein was capable of DNA extension activity with magnesium and manganese (Supplementary Figure S2B). It was proficient at primer extension in the presence of dNTPs on every nucleic acid substrate combination (Supplementary Figure S2C) but exhibited very limited strand-displacement activity (Supplementary Figure S2D). The primer synthesis activity was not dependent on the presence of NTPs (Supplementary Figure S2E) and was only supported by manganese (Supplementary Figure S2F). The initiation of *de novo* synthesis was most efficient on templates containing a CC sequence (Supplementary Figure S2G). The RT domain catalytic mutant (D532N, D533N) was inactive. Although RT-dependent priming was most efficient on CC sequences *in vitro*, other factors may influence the sequence preference of *Mm* RT domain *in vivo*, e.g. cellular concentrations of nucleotides, sequence context, secondary structures, etc. which could significantly change or abolish RT’s bias for specific RNA template sequences. Therefore, it is not surprising that no preference for protospacer sequence, including 15 bp of flanking sequence on each protospacer, was observed (Note: spacers in the CRISPR array are (usually) derived from fragments of viral or plasmid genetic information termed protospacers.) (16). However, we can’t exclude the possibility that the synthesized prespacers, derived from protospacers, are significantly processed before their integration into the CRISPR array and therefore this information has been lost in the sequencing analyses. Nonetheless, *Mm* RT domain's clear preference for CC sequences *in vitro* was taken advantage of when designing RNA templates for the modified prespacer integration assays, described below.

Next, we utilized a 5’ FAM-labelled *Mm*CRISPR array and a variety of 5’ Cy5/Cy3-labelled DNA or RNA substrates (Supplementary Figure S3A), to visualize products of prespacer integration assays. Under conditions with manganese and without dNTPs, we observed integration of ssDNA, dsDNA and the DNA strand of an RNA-DNA heteroduplex into the *Mm*CRISPR array by *Mm*Cas6-CART-Cas1–Cas2 complex. However, RNA integration under the same conditions, either stand-alone or in a hetero-duplex with DNA, was very inefficient (Supplementary Figure S3B) suggesting that DNA synthesis is requisite for integration of RNA-derived prespacers (16). In assay conditions with magnesium and with or without dNTPs, integration was observed the only for ssDNA (Supplementary Figure S3C) and in the presence of dNTPs a small fraction of ssDNA and ssRNA prespacers was extended. However, both ssDNA and ssRNA were integrated in presence of manganese and dNTPs (16) (Supplementary Figure S3D, S3E) and a significant fraction of both prespacers was extended. RT domain catalytic mutant (D532N, D533N; RT M) did not extend these protospacers, indicating that the RT domain was responsible for the non-specific extension of ssDNA and ssRNA prespacers. This enabled the integration of ssRNA prespacers, extended by dNTPs, into the *Mm*CRISPR array (Supplementary Figure S3E) and showed that *Mm*Cas6-CART-Cas1–*Mm*Cas2 complex requires deoxynucleosides on the 3’ end of the prespacer for the integration. It was previously suggested, that RT domain is extending the 3’ end of the ‘nicked’ CRISPR array DNA strand and copying the repeat and integrated ssRNA (16), which could be attributed to strand displacement synthesis. However, we did not observe extension of the 3’ end of the ‘nicked’ CRISRPR array DNA strands (Supplementary Figure S3C), possibly due to the limited strand displacement activity of *Mm*Cas6-CART-Cas1 (Supplementary Figure S2D), consistent with activities found in other Cas6-CART-Cas1 proteins (19). These findings indicate that *Mm*Cas6-CART-Cas1 is unlikely to be involved in strand-displacement synthesis after integration of ssRNA into CRISPR arrays, as was previously proposed (16).

**Figure 4.**
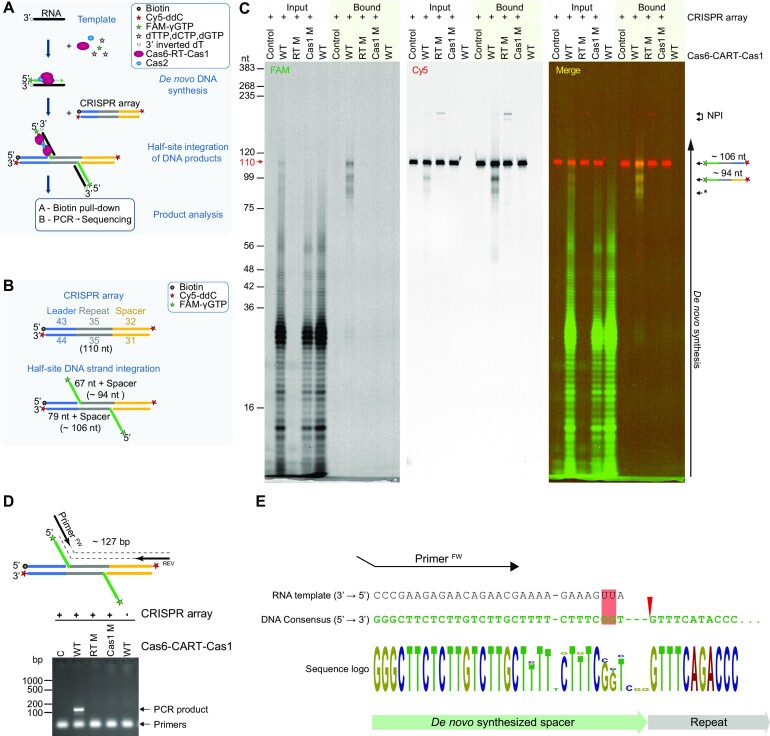
Primase activities of CART facilitates integration into CRISPR arrays. (**A**) Graphical scheme of an *in vitro* primed prespacer integration assay. Integration products were either pull-down via biotin and separated on denaturing PAGE or analysed by PCR and sequencing. (**B**) Graphical representation of expected products of the *in vitro* primed prespacer integration assay using *Mm*CRISPR array. The length of the expected products is indicated. (**C**, **D**) *Mm*Cas6-CART-Cas1 integrates *de novo* synthesized DNA into a *Mm*CRISPR array. Integration products in input and streptavidin beads bound fractions (bound) were separated on urea-PAGE (panel C) or the integration products were PCR amplified (panel D). RT M indicates MmCas6-CART-Cas1 D532N, D533N mutations in the RT domain and Cas1 M is for Cas1 mutation in *Mm*Cas6-CART-Cas1 H855A, E870A. NPI, non-prespacer integrations; Nt, nucleotides; Bp, base pairs; Black star (*), integration of shorter primed DNA products into CRISPR array; C, control without protein. Size markers are indicated on the left of gels. The red arrow on the left side of the gel (panel C) indicates size of Cy5-labelled CRISPR array without integration. (**E**) Sequences of integrated spacers in *Mm*CRISPR arrays. Black arrow indicates forward primer (Primer^FW^) used for PCR amplifications. Red triangle indicates position of prespacer integration into the *Mm*CRISPR array. Pink rectangle indicates imprecise reverse transcription of the ssRNA template due to the lack of dATP in the reaction and Wobble base pairing. Sequence logo height indicates the nucleotide frequency in the sequence alignment.

However, the *Mm*Cas6-CART-Cas1—*Mm*Cas2 complex could potentially utilize its RT-dependent primase activity to synthesise a DNA prespacer, originating from ssRNA, and integrate it into the CRISPR array. To test this hypothesis, we used a modification of the prespacer integration assay (Figure 4A, B), in which *Mm*Cas6-CART-Cas1—*Mm*Cas2 complex was preincubated with ssRNA template, nucleotides (dCTP, dGTP, dTTP and γ-phosphate FAM-labelled GTP (FAM-γGTP)) and manganese to allow *de novo* synthesis of 5’ end FAM-labelled DNA. To prevent non-specific extension and integration, the ssRNA template was chain terminated with an inverted dTTP on the 3’ end. *Mm*CRISPR array, biotin-labelled on its 5’ end of its leader sequence and chain terminated and Cy5-labelled on both 3’ ends (Cy5-dideoxycytidine) (Figure 4A), was added to initiate the prespacer integration. After the integration, the biotinylated CRISPR array was bound to streptavidin beads to selectively purify *de novo* synthesized FAM-labelled DNA prespacers integrated into *Mm*CRISPR arrays, and products were separated on denaturing gels (Figure 4C). Successful *de novo* synthesis was observed in the input fraction (FAM channel) for *Mm*Cas-CART-Cas1 wild-type (WT), and also with the Cas1 domain mutant (H855A, E870A; Cas1 M), but not with the RT domain mutant (RT M). *De novo* synthesized DNA integrated into the *Mm*CRISPR array can be observed as an overlap of the green signal (*de novo* synthesized FAM-labelled DNA) and red signal (Cy5-labelled *Mm*CRISPR array) in the bound fraction (Figure 4C) at predicted lengths of the integrated products (∼94 and ∼106 nt; Figure 4B). No integration was observed with the Cas1 mutant (Figure 4C). Together, these results establish that the Cas1-dependent integration into CRISPR array of *de novo* synthesized DNA strands copied from RNA templates is dependent on the RT primase activity.

These results were validated by PCR analyses to confirm that products from the CRISPR integration assays represent *bona fide* RNA-derived sequences (Figure 4D). Primers complementary to the 3’ end of the RNA template and the 3’ spacer end of the *Mm*CRISPR array were used to amplify the integration products (Figure 4D, top panel). Notably, only reactions containing ssRNA template, *Mm*CRISPR array and *Mm*Cas6-CART-Cas1 WT produced amplicons of the expected size (∼127 nt), confirming the specific integration of *de novo* synthesized DNA (Figure 4D, bottom panel). These amplicons were not observed with mutant proteins (RT M and Cas1 M) or when the CRISPR array was omitted. Sub-cloning of integration products after PCR amplification and sequencing of individual colonies confirmed that the consensus sequence of the spacers integrated into the leader-repeat junction of the *Mm*CRISPR array was complementary to the RNA template (Figure 4E, Supplementary Figure S4). The new spacers integrated into the *Mm*CRISPR array varied in length and sequence, which corelates with the observation of variable length of *de novo* synthesized products (Figure 4C). Together, these findings establish that the primase activity of the RT domain of *Mm*Cas6-CART-Cas1 is required to reverse transcribe ssRNA templates into new DNA strands, which are then efficiently integrated into the *Mm*CRISPR arrays by Cas1–Cas2.

### Crystal structure of the RT domain of *Ca*CART-CAPP

**Figure 5.**
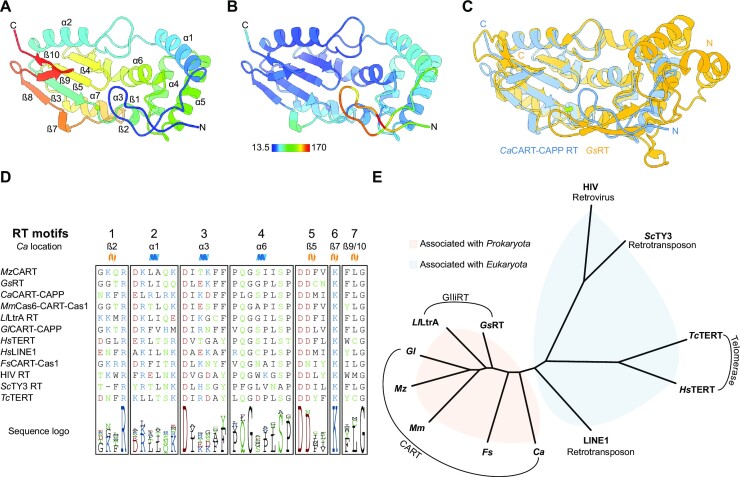
Crystal structure of the RT domain of *Ca*CART-CAPP. (**A**) Crystal structure of the RT domain of *Ca*CART-CAPP (8bgj). Rainbow colour denotes primary sequence and secondary structures are annotated. (**B**) Structure of *Ca*CART-CAPP RT domain coloured by *B*-factor. (**C**) Superposition of RT domain of *Ca*CART-CAPP (8bgj) (blue) with *Gs*RT (6ar1) (orange). (**D**) Sequence alignment of selected CART RT domains. The sequences are trimmed to only show conserved sequence motifs of the RT superfamily (1–7). Protein accession numbers in descending order: WP_035075942, WP_053413546, CCJ33120, ADZ89953, WP_081213830, ASS88148, NP_937983, AAB59368, WP_055226073, P04585, Q99315, NP_001035796. (**E**) Unrooted phylogenetic tree of selected RT domains. RTs association with domains of life is highlighted, prokaryotic organisms (orange), eukaryotic organisms—including viruses that infect them - (blue).

To better understand the architecture of CARTs, we elucidated the crystal structure of RT domain of *Ca*CART-CAPP at 1.63 Å (Figure 5A), crystallised in space group P3_2_ with 3 monomers in the asymmetric unit (Table 1). The architecture of the RT catalytic core is composed of a ‘palm’ subdomain with an RNA Recognition Motif like (RRM-like) fold, which contains catalytic acidic residues bound to a single metal ion (manganese coordinated by D73, I74 and D154) and ‘fingers’ subdomain, adhering to the canonical ‘hand’ analogy for replicative polymerases. The N-terminal part of the ‘fingers’ subdomain is highly dynamic, reflected by the high B-factors in the modelled residues (average of 122.0 Å^2^ for atoms in the first 20 residues, compared to 40.4 Å^2^ for the rest of the monomer) (Figure 5B). The structure of *Ca*CART-CAPP RT domain was elucidated by molecular replacement using a bacterial *Geobacillus stearothermophilus* GsI-IIC intron RT (*Gs*RT) from the GIIiRT protein family (31). Both structures are remarkably similar, with a root-mean-square deviation (RMSD) of 2.1 Å across 190 aligned residues (Figure 5C). The catalytic core of GIIiRTs contains three major extensions, which differentiates them from retroviral RTs, including an N-terminal extension (NTE), Motif 2 extension (RT2e) and Motif 3 extension (RT3a) (31). *Ca*CART-CAPP RT domain lacks an NTE, which is likely contributing to the higher flexibility of the N-terminus. The alignment of selected members of the RT protein superfamily shows the conserved RT motifs (Figure 5D) among different phylogenetic branches (Figure 5E).

**Table 1.** X-ray data collection and structure refinement statistics

	CaCART-CAPP
	RT domain
Deposition ID (PDB)	8BGJ
**Data collection**	
Wavelength (Å)	0.96864
Space group	*P*32
Cell parameters	
a, *b*, *c* (Å)	96.9, 96.9, 65.4
α, β, γ (°)	90, 90, 120
Resolution limits (Å) (high-resolution bin)*	
83.94–1.63	
(1.66–1.63)	
Completeness (%)	99.6 (99.5)
Multiplicity	3.1 (3.1)
CC-half	0.992 (0.318)
I/sigma	9.0 (0.5)
Rmerge	0.076 (1.184)
**Refinement**	
Protein monomers per	
asymmetric unit	3
Total nonhydrogen atoms	5503
Water molecules	331
Rcryst (%)	18.30
Rfree (%)	21.88
Ramachandran analysis (%)	
Most favoured	93.75
Outliers	2.03
Rmsds	
Bonds (Å)	0.007
Angles (°)	0.95
Planes (Å)	0.008
Mean atomic *b* value (Å^2^)	41.23

### Conservation of DNA priming in other RT superfamily members

**Figure 6.**
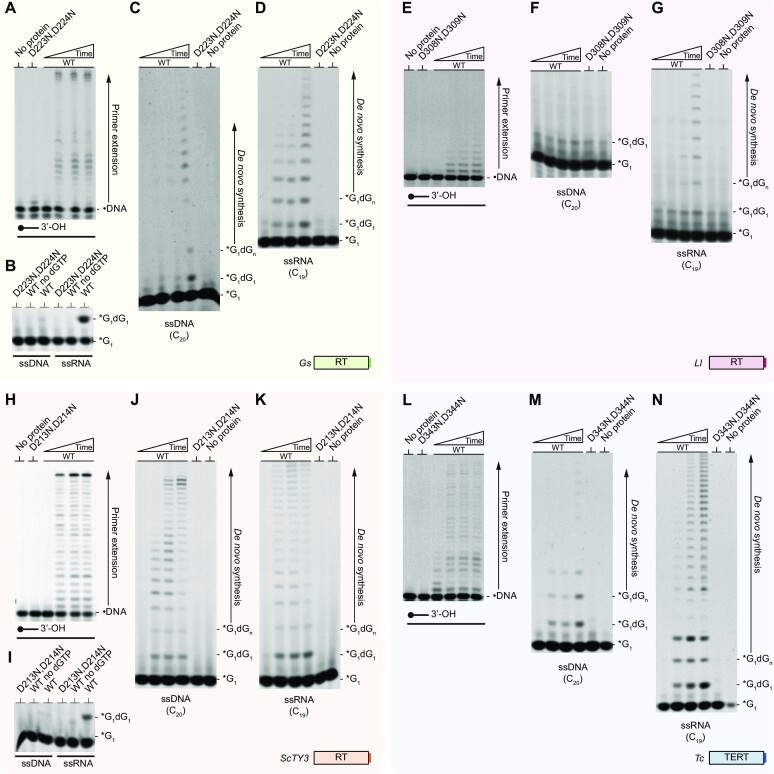
Conservation of DNA priming in other RT superfamily members. (**A**) DNA polymerase activity of *Gs*RT. Time course with 50 nM DNA duplex (oPK404 + oPK405), 50 nM protein, Mn^2+^ and dNTPs. (**B**) Dinucleotide synthesis of *Gs*RT. 1 μM substrate (DNA, oKZ153; RNA, oMZ11) was incubated with 1 μM protein, γ- phosphate FAM-labelled GTP and dGTP. (**C, D**) *Gs*RT prefers to prime on RNA template. Time course of the primase activity of *Gs*RT with 1 μM DNA (oMZ13) (panel C) or RNA template (oMZ27) (panel D), 1 μM protein, γ-phosphate FAM-labelled GTP, dGTP and Mn^2+^. D223N, D224N presents catalytic mutant of *Gs*RT. (**E**) Polymerase activity of *Ll*LtrA RT domain. Time course with 50 nM 5’ end FAM-labelled DNA primer (oPK405) annealed with RNA template (oPK406) 1 μM protein, Mn^2+^ and dNTPs. (**F, G**) *Ll*LtrA RT is an RNA-dependent primase. Time course of the primase activity of *Ll*LrtA RT domain with 1 μM DNA (oMZ13) (panel F) or RNA template (oMZ27) (panel G), 1 μM protein, γ-phosphate FAM-labelled GTP, dGTP and Mn^2+^. D308N, D309N presents catalytic mutant of *Ll*LtrA RT. (**H**) Polymerase activity of *Sc*Ty3 RT domain. Time course with 50 nM 5’ end FAM-labelled DNA primer (oPK405) annealed with RNA template (oPK406), 1 μM protein, Mg^2+^ and dNTPs. (**I**) Dinucleotide synthesis of *Sc*Ty3 RT domain. 1 μM substrate (DNA, oKZ153; RNA, oMZ11) was incubated with 1 μM protein in presence of γ-phosphate FAM-labelled GTP, dGTP and Mn^2+^. (**J, K**) *Sc*Ty3 RT is efficient DNA and RNA-dependent primase. Time course of the primase activity of *Sc*Ty3 RT domain with 1 μM DNA (oMZ13) (panel J) or RNA template (oMZ27) (panel K), 1 μM protein, γ-phosphate FAM-labelled GTP, dGTP and Mn^2+^. D213N, D214N presents catalytic mutant of *Sc*Ty3 RT. (**L**) DNA polymerase activity of *Tc*TERT RT domain. Time course experiment with 50 nM DNA duplex (oPK404 + oPK405), 50 nM protein in presence of Mg^2+^ and dNTPs. (**M, N**) *Tc*TERT RT domain is DNA- and RNA-dependent primase. Time course of the primase activity of 1 μM *Tc*TERT with 1 μM DNA (oMZ13) (panel M) or RNA template (oMZ27) (panel N) in presence of γ-phosphate FAM-labelled GTP, dGTP and Mn^2+^. D343N, D344N presents catalytic mutant of *Tc*TERT RT. Products of polymerase and primase assays are resolved on 10% and 20% TBE-Urea-PAGE gels respectively (panels A–N). WT, wild type; *G, γ-phosphate FAM-labelled GTP; •DNA, FAM-labelled DNA primer. Time course–polymerase assay (panels A, E, H and L): 0, 5, 10 and 30 min.; Time course–primase assay (panels B–D, F–G, I–K and M–N): 0, 2, 10 and 30 min; No protein control and catalytic mutants of RT domains were incubated for 30 min.

The close sequence and structural relationships of the core catalytic residues prompted us to investigate possible primase activities among other major phylogenetic branches of the RT superfamily. GIIiRTs comprise the largest RT family in prokaryotes and play major roles in the life cycle of mobile genetic elements (MGEs), from which they arise. We selected *Gs*RT, a prototypical stand-alone GIIiRT, that operates as part of a group II intron retrotransposon (37). Full length *Gs*RT preferentially extends DNA primers on DNA and RNA templates with dNTPs (Figure 6A, Supplementary Figure S5A) but extension with NTPs is less efficient (Supplementary Figure S5B). Similar to CART primase activities, *Gs*RT displays robust dinucleotide synthesis with preference for RNA templates (Figure 6B), which results in weaker DNA-dependent priming (Figure 6C) when compared to RNA-dependent priming (Figure 6D). The initiation of *de novo* DNA synthesis is not dependent on the presence of NTPs (Supplementary Figure S5C). *Gs*RT can efficiently form different dinucleotides, showing a broader substrate specificity for the templated initiation of synthesis than observed for *Ca*CART-CAPP RT domain (Supplementary Figure S5D). The primase activity is supported by manganese and cobalt (Supplementary Figure S5E) at temperatures up to 50°C (Supplementary Figure S5F). The RT domain catalytic mutant (D223N, D224N) didn’t possess any activity. In contrast to CARTs, *Gs*RT exhibited a much stronger preference for RNA substrates. We hypothesise that, given that the life cycle of group II introns involves reverse transcription of an intron RNA during retrotransposition, DNA-dependent synthesis may not be required for its physiological functions.

Other mobile group II introns encode RTs that contain an additional endonuclease domain (EN), proposed to produce 3’ ends that act as initiation primers for their extension activity. We purified the RT domain of *Ll*LtrA (aa 1–472) from *Lactococcus lactis* (*Ll*) (38) lacking the EN domain and observed that, besides its primer extension activity (Figure 6E), it also possesses RNA-dependent primer synthesis activity (Figure 6F, G), not observable in RT domain catalytic mutant (D308N, D309N). Such *de novo* DNA synthesis activity can provide an alternative, or even complementary, mode of priming that assists in the replication of MGEs.

Budding yeast (*Saccharomyces cerevisiae*) Ty3 LTR-retrotransposon (*Sc*Ty3) encodes a RT required for its replication, which is more divergent when compared to bacterial RTs. To determine if it exhibits catalytic activities similar to CARTs or GIIiRTs, we purified the RT domain of *Sc*Ty3 (aa 1–339) (*Sc*Ty3 RT) with a C-terminal MBP fusion. We observed DNA-dependent DNA polymerase activity (Figure 6H), utilizing magnesium, manganese or cobalt for primer extension (Supplementary Figure S6A). We observed extension of DNA, but not RNA, primers (Supplementary Figure S6B). This is contrary to a previous report (39), which may be due to the absence of a nucleocapsid protein (NCp9) to facilitate extension of RNA primers (40,41). Similar to *Gs*RT, *Sc*Ty3_RT displays dinucleotide synthesis (Figure 6I), primer synthesis on DNA (Figure 6J) and RNA templates (Figure 6K), with preference for RNA. This activity is only facilitated by manganese (Supplementary Figure S6C) and formation of dinucleotides was proficient for all combinations starting with a guanosine (Supplementary Figure S6D). The RT domain catalytic mutant (D213N, D214N) was inactive. In conclusion, *Sc*Ty3 RT domain possesses both DNA and RNA-dependent DNA primase activity and is very likely to be involved in the replication cycle of the *Sc*Ty3 retrotransposon.

Discovering the unexpected *de novo* priming activities of RTs encoded within eukaryotic retrotransposons led us to next investigate the extension activities of eukaryotic telomerases, which contain a catalytic RT domain at their core. We chose to study the RT domain of red beetle *Tribolium castaneum* telomerase (*Tc*TERT). *Tc*TERT RT domain displayed DNA primer extension activity on DNA templates (Figure 6L). Similar to GIIiRT and *Sc*Ty3 RT, telomerase's RT domain also possesses DNA-dependent (Figure 6M) and RNA-dependent primase activity (Figure 6N), with an apparent preference for RNA templates. The physiological roles(s) of the primase activity of telomerases’ RT domains remains to be established.

**Figure 7.**
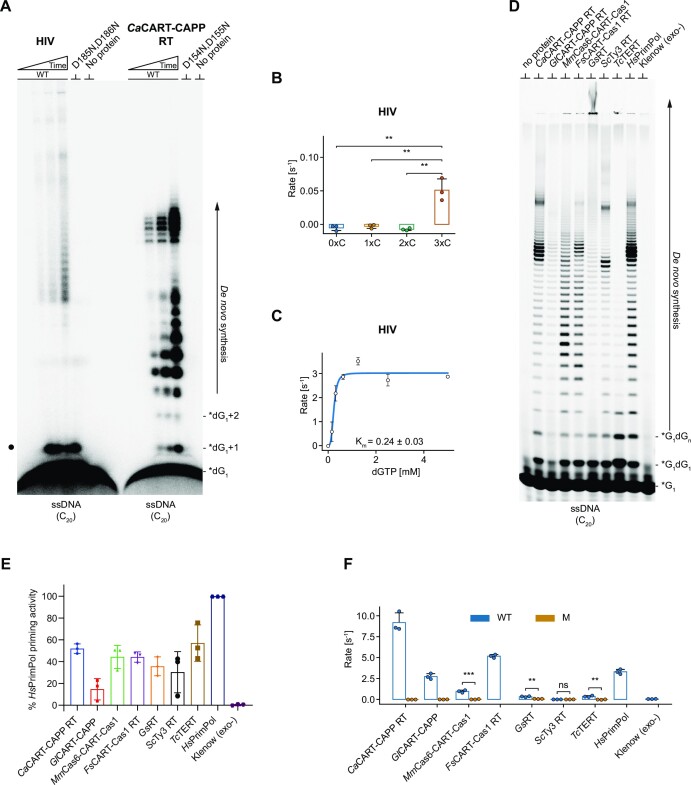
Primase activity of HIV RT and comparison of primase activities of RTs with HsPrimPol. (**A**) Time course of HIV RT (p66–p51 heterodimer) primase activity, compared to CaCART-CAPP RT domain. 1 μM protein was incubated with α-32P-labelled dGTP and dGTP, with 1 μM ssDNA (oMZ13). The gel was developed for 1 hour on BAS-MS imaging plate (Fujifilm) and scanned on IP stage of FLA 5100 (Fujifilm). D185N, D186N represents catalytic mutant of both subunits of HIV RT. D154N, D155N represents catalytic mutant of *Ca*CART-CAPP RT. Black spot indicates position of synthesized dinucleotides. (**B**) Sequence requirement of HIV RT for the primase activity on ssDNA. 250 nM protein incubated with 1 μM ssDNA (oKZ150, oKZ294, oKZ153, oMZ2), dGTP and dATP. DNA synthesis was measured using a dsDNA intercalating fluorescent dye-based assay and statistical analysis used two-tailed independent *t*-test. **P-value < 0.01. (**C**) Initial reaction rates of 250 nM HIV RT with increasing dGTP and 1 μM homopolymeric cytosine DNA substrate (oMZ13), fitted to Hill-Langmuir equation and with Km calculated. Measured using a dsDNA intercalating fluorescent dye-based assay. (**D**) Comparison of RTs, *Hs*PrimPol and Klenow (3'→5' exo-) (NEB, M0212S) primase activities using gel-based primase assay. 1 μM protein, Mn^2+^, dGTP, γ-phosphate FAM-labelled GTP and 1 μM ssDNA template (oMZ13) was incubated at 20°C for 30 min. (**E**) Quantification of the gel-based primase assay in panel D. (**F**) The comparison of RTs priming activities. The intercalating fluorescent dye-based primase assay with 250 nM protein on 1 μM ssDNA (oKZ148, C_20_T_20_) and statistical analysis with two-tailed independent *t*-test. **P-value < 0.01; ***P-value < 0.001; ns, not significant. Products of gel-based primase assays are resolved on 20% TBE–urea–PAGE gels (panels A, D). Template and product sequences: 5’ → 3’; WT, wild type; M, RT mutant; *dGTP, [α-32P] 10 mCi/ml; *G, γ-phosphate FAM-labelled GTP; Time course–primase assay (panel A, D): 0, 2, 10 and 30 min; no protein control and catalytic mutants of RT domains were incubated for 30 min.

Retroviral RTs were next investigated, choosing to study the p66 - p51 heterodimer from human immunodeficiency virus (HIV RT). Initially, we did not observe any significant primase activity in gel-based primase assays. However, it was later discovered that this resulted from an inability of HIV RT to incorporate fluorescently labelled nucleotides during primer synthesis. To circumvent this issue, we used radiolabelled dGTP in gel-based primase assays and observed robust *de novo* DNA primer synthesis of HIV RT (Figure 7A). The primase activity of HIV RT was unexpectedly stronger on homopolymeric ssDNA, than ssRNA (Supplementary Figure S7A). This led to further investigation of its substrate preferences using an intercalating fluorescent dye-based primase assay. HIV RT initiated *de novo* DNA synthesis only on ssDNA containing at least three consecutive cytosines (Figure 7B), which notably correlates with the three cytosines in the tRNA primer binding region of the viral RNA (42). The affinity for dGTP on a homo-polymeric ssDNA template (C_20_T_20_) was approximated by measuring its K_m_ in the sub-millimolar ranges (Figure 7C). Further studies are required to establish whether *de novo* primer synthesis occurs during viral replication *in vivo*.

Next, we compared the primase activities of all RTs from this study with full-length human PrimPol protein (*Hs*PrimPol), a member of Prim-Pol superfamily with established primase activities (43,44). The results from two different primase assays are shown here. First, a gel-based primase assay (Figure 7D) and its quantification (Figure 7E) and, second, an intercalating fluorescent dye-based primase assay (Figure 7F). Klenow fragment (3'→5' exo-), an active polymerase lacking primase activity was used as a negative control. The Klenow fragment had no detectable primase activity in the first assay, with either with γ-phosphate FAM-labelled GTP (Figure 7D, E) or FAM-labelled dCTP (Supplementary Figure S7B), although it was able to incorporate FAM-labelled dCTP in a primer extension assay (Supplementary Figure S7C). Similarly, no primase activity of Klenow fragment was detected in the second assay (Figure 7F). All tested RTs showed level of primase activities comparable to *Hs*PrimPol in the gel-based assay (Figure 7D, E). We additionally show the primase activity of *Fusicatenibacter saccharivorans Fs*CART-Cas1 RT domain, to exemplify a conservation of such activity in a protein domain fusion, other than shown previously. Note, *Ll*LtrA RT and HIV RT were excluded from the comparison as *Ll*LtrA RT showed no detectable primase activity on ssDNA templates (Figure 6F) and HIV RT doesn’t incorporate FAM-labelled nucleotides. In the intercalating fluorescent dye-based primase assay, most of RTs showed primase activities either comparable to *Hs*PrimPol, or at least significantly more than their catalytic mutants (Figure 7F, Supplementary Figure S7D). We attribute the lower observable activities for some of RTs to the limitation of the second assay, which can only use ssDNA substrates, and some RTs having much stronger activities on ssRNA, e.g*. Ll*LtrA RT and *Sc*Ty3 RT. In summary, the primase activities of most RTs are comparable with a known exemplar primase, indicating that these observations are not artefactual, and thus establishing that RTs form a group of replicases capable of *de novo* primer synthesis *in vitro*.

### Insights into the conservation of DNA priming mechanisms

**Figure 8.**
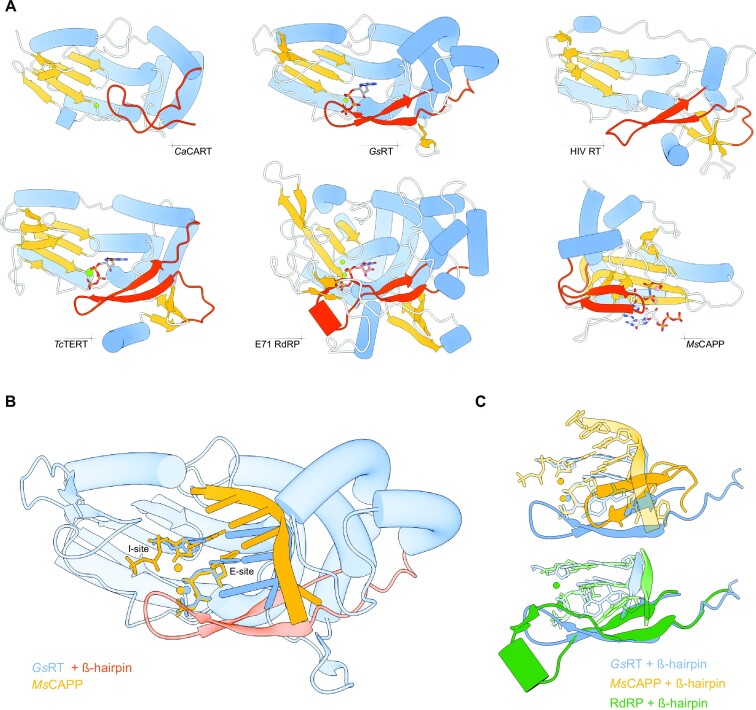
Insights into the conservation of DNA priming mechanisms. (**A**) Comparison of the RRM-like fold of RTs (*Ca*CART-CAPP RT 8bgj, *Gs*RT 7k9y, HIV RT 6hak, *Tc*TERT 7kqn), RdRP (E71 7w9s) and Prim-Pol (*Ms*CAPP 7p9y). Orange, β-sheet; blue, α-helix; red, β-hairpin. (**B**) Superposition of nucleotides and templates from the active sites of *Gs*RT (primer-template ternary complex; 7k9y) in blue and *Ms*CAPP (primer initiation ternary complex; 7p9y) in orange. Red, β-hairpin from *Gs*RT. (**C**) Superposition of nucleotides and templates from active sites of *Gs*RT (7k9y)—blue, *Ms*CAPP (7p9y)—orange and E71 RdRP (7w9s)—green. Only the β-hairpins from their protein structures are shown. Reverse transcriptases (RTs) are replicative enzymes that copy RNA into DNA and undertake roles, including viral replication, retrotransposition and telomere maintenance. The initiation of RT synthesis activities is usually dependent on the presence of a primer. The current dogma proposes that a variety of indirect, RT-independent, priming mechanisms instigate synthesis. However, this study establishes that CRISPR-associated RTs (CARTs) are capable of priming DNA synthesis from scratch, which enables the capture of foreign genetic material for storage in CRISPR arrays. The authors also report that other notable RT family members, including retrotransposon RTs, telomerase and retroviral RT are, surprisingly, able to directly catalyze primer synthesis. These findings significantly alter our understanding of priming mechanisms utilised by RTs in various biological pathways.

CARTs and GIIiRTs share a number of unique extensions to their RT domain catalytic cores and structural equivalents of these elements are found in viral RNA-dependent RNA polymerases (RdRPs) (31) or telomerases, exemplified by their high structural homology to *Ca*CART-CAPP RT domain (Supplementary Table S7). However, these extensions appear to be dispensable for primer synthesis, because the RT domain of *Sc*Ty3 is lacking these extensions and still retains its primase activity. It was proposed that these RT motif extensions increase the overall processivity of these RTs, by increasing the number of contacts with substrates (31,45,46). The most intriguing observation is the positioning of a β-hairpin in the ‘fingers’ subdomain, with structural equivalents found within RTs, telomerases, viral RdRPs and Prim-Pols (Figure 8A). Deletion of the β-hairpin from *Ca*CART-CAPP ablates its polymerase and primase activities (Supplementary Figure S8A, S8B), similar to replacing residues in the equivalent region of *Marinitoga piezophila* CAPP (*Mp*CAPP) or *Hs*PrimPol implicated in nucleotide binding (47). This suggests that having this mutually conserved structural element may be a prerequisite for proficient primer synthesis. This structural conservation is also supported by previous bioinformatic analyses of RRM-like fold replicases (11), suggesting an evolutionary model for replicative enzymes containing an RRM-like fold.

To better understand if conserved structural elements are employed by RRM-like fold based replicases to facilitate primer synthesis, we superposed the crystal structures of a *Gs*RT ternary complex (31,45) onto a *Marinitoga sp1137* CAPP (*Ms*CAPP) primer initiation complex (47) (Figure 8B). This comparison shows the positioning of initiating (I-site) and elongating nucleotides (E-site), metal ions and templating nucleic acid within the active site of two structurally divergent proteins. Despite the major differences in the core protein structure, the positioning of the nucleotides is remarkably similar, as well as is the positioning of the β-hairpin of RTs when compared to equivalents found in PrimPols and RdRPs (Figure 8C). Similar to the *Ms*CAPP priming complex, most of *Gs*RT’s predicted major contacts are made with the template strand and the E-site nucleotide (Supplementary Figure S8C). However, limited interactions are evident with the I-site nucleotide, which is likely bound in the active site by base pairing, π-stacking interactions with neighbouring bases, and electrostatic interactions with the A-site metal ion, as previously proposed for CAPP (47).

## DISCUSSION

A common mechanistic feature of nearly all DNA and RNA polymerases is the requirement for a 3’ hydroxyl moiety to act as the primary nucleophile requisite for phosphodiester bond formation with the incoming nucleotide triphosphate, which extends the newly synthesizing strand. Typically, this crucial moiety is provided by the synthesis of a short primer strand, which is subsequently extended by a polymerase. During canonical DNA replication, primers are made by a bespoke class of replicases called DNA primases that initiate primer synthesis *de novo*. In bacteria, DnaG primases from the TOPRIM family undertake this role. In eukaryotes and archaea, primases belonging to the Primase-Polymerase (Prim-Pol) superfamily perform this priming function (48). In contrast, many RNA-dependent RNA polymerases involved in viral replication catalyse *de novo* primer synthesis prior to extension (49), eliminating the requirement for an additional bespoke primase. However, for reverse transcription the currently accepted model is that RTs use a diverse range of non-canonical priming mechanisms, including usage of the 3’ termini of tRNAs and endonuclease products to start *de novo* DNA synthesis (14,42). However, this current study establishes that RTs have retained a primordial ability to prime synthesis on RNA, as well as DNA templates. These findings have significant implications for our understanding of the *modus operandi* of RTs, which operate in a diverse range of DNA synthesis pathways across all domains of life and are also widely used in a range of molecular biology applications.

Prokaryotic RTs operate during the adaptation stages of a subset of type III CRISPR-Cas pathways, which facilitate the acquisition of foreign nucleic acids. A notable feature of Type III CRISPR-Cas pathways is their ability to acquire new spacers from both RNA and DNA sources *in vivo*, although how this is achieved has remained obscure. Here we establish that CRISPR-associated RTs primase activity plays direct roles in the acquisition of nucleic acids sequences during spacer adaptation. We demonstrate that CARTs can directly prime DNA synthesis on RNA / DNA templates to produce DNA prespacers, which are subsequently integrated by Cas1–Cas2 into CRISPR arrays. Given that Cas1–Cas2 is very inefficient at integrating RNA prespacers, CARTs innate ability to directly prime off RNA templates, enabling them to be reverse transcribed into DNA, significantly enhances the efficiency of spacer acquisition of new sequences from foreign sources of RNA. Previous studies have reported that some CRISPR-Cas systems rely on host nucleases, e.g. RecBCD complex, to assist in the production of prespacers for naïve adaption purposes (50). These nucleases cleave invading DNA into short fragments that are further processed into prespacers for subsequent integration by Cas1-Cas2. We propose that CARTs capacity to directly prime synthesis on a diverse variety of nucleic acid templates provides an alternative and highly flexible mechanism to ‘convert’ foreign DNA / RNA sequences into prespacers for naïve spacer acquisition. How CART-dependent prespacer synthesis is coordinated with its partners during CRISPR-Cas adaptation and how these products are subsequently processed for integration *in vivo* remains to be established. It remains unclear why CART-CAPP fusion proteins studied here have retained an inactive PP domain. A likely scenario is, the RT domain was acquired through insertion into the host genome by various retroelements, as suggested previously (21). The acquisition of RT may have provided CRISPR-Cas systems with more ‘flexibility’ to acquired spacers from both RNA and DNA substrates, thus potentially making the PP domain redundant and prone to loss-of-function mutagenesis. Nonetheless, our results reveal that the DNA synthesis activity is provided by the RT domain and we also show the functional relevance for such activities in a CRISPR-Cas system.

The discovery that CRISPR-associated RTs initiate *de novo* DNA synthesis was totally unexpected, but it suggested that other RTs may also possess equivalent capacities to directly prime DNA synthesis. Starting with the closely related prokaryotic group II retrotransposon-encoded RTs, we show that both stand-alone *Gs*RT and even those possessing an endonuclease domain, like *Ll*LtrA, display an intrinsic ability to prime on RNA templates. We subsequently extended these findings to the eukaryotic LTR-retrotransposon encoded RT (*Sc*Ty3 RT) by establishing that it is also proficient in *de novo* DNA synthesis and exhibit a broad template specificity. Finally, we demonstrate that a telomerase RT involved in telomere maintenance and retroviral RT from HIV, also possesses primase activity. The current dogma proposes that RT-dependent replication mechanisms of MGEs, telomeres and retroviruses rely on a range of alternative priming mechanisms to initiate DNA synthesis (14). However, this study unequivocally establishes that a variety of different RTs directly catalyse primer synthesis *in vitro*, and that this activity plays a specific role in CRISPR-Cas spacer acquisition. All of the priming activities of RTs described in this study were observed exclusively *in vitro* so the next important step is to investigate whether these enzymes can also prime *in vivo* to determine if RT-dependent priming is biologically relevant. Follow-up studies are also necessary to establish how *de novo* RT priming mechanisms operate alongside the repertoire of alternative priming mechanisms proposed to initiate DNA synthesis in a diverse range of other RT-dependent pathways, from viral replication to telomere maintenance.

## Supplementary Material

gkad478_Supplemental_Files

## Data Availability

All data are provided in full in the results section and the Supplementary Information accompanying this paper. Atomic coordinates and structure factors have been deposited in the Protein Data Bank under the accession code 8BGJ.
