# Supplementary material for: Reverse transcriptases prime DNA synthesis
Source: Nucleic Acids Res. 2023 Jun 6;51(14):7125–42. doi: 10.1093/nar/gkad478 (PMC10415136; doi:10.1093/nar/gkad478)
Supplement: gkad478_Supplemental_Files [file gkad478_supplemental_files.zip › Supplemental Information_final.pdf]

Supplemental Information

Reverse transcriptases prime DNA synthesis

Matej Zabrady[1+], Katerina Zabrady[1+], Arthur W-H Li[1], and Aidan J. Doherty[1*]

[1] Genome Damage and Stability Centre, School of Life Sciences, University of Sussex, Brighton BN1 9RQ, UK

+ These authors contributed equally to this work

[*] Corresponding author: Aidan Doherty, email: ajd21@sussex.ac.uk

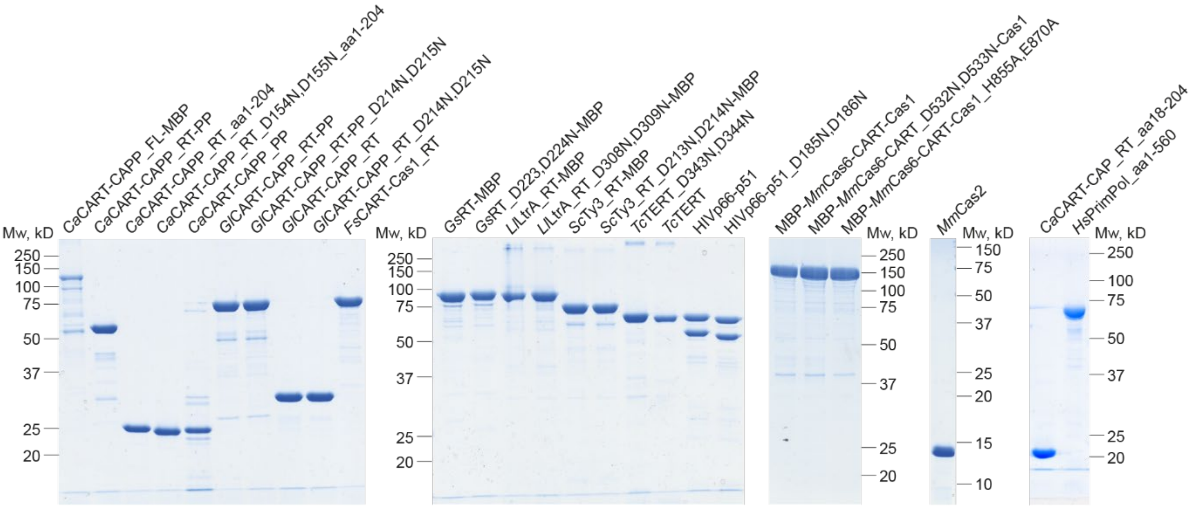

Figure S1: Purified proteins used in this study

2 µg of purified proteins were run on 12% SDS-PAGE, except *Mp*Cas2 which was run on 17% SDS-PAGE, and Coomassie stained (InstantBlue®, Abcam). MBP indicates fusion with Maltose-binding protein.

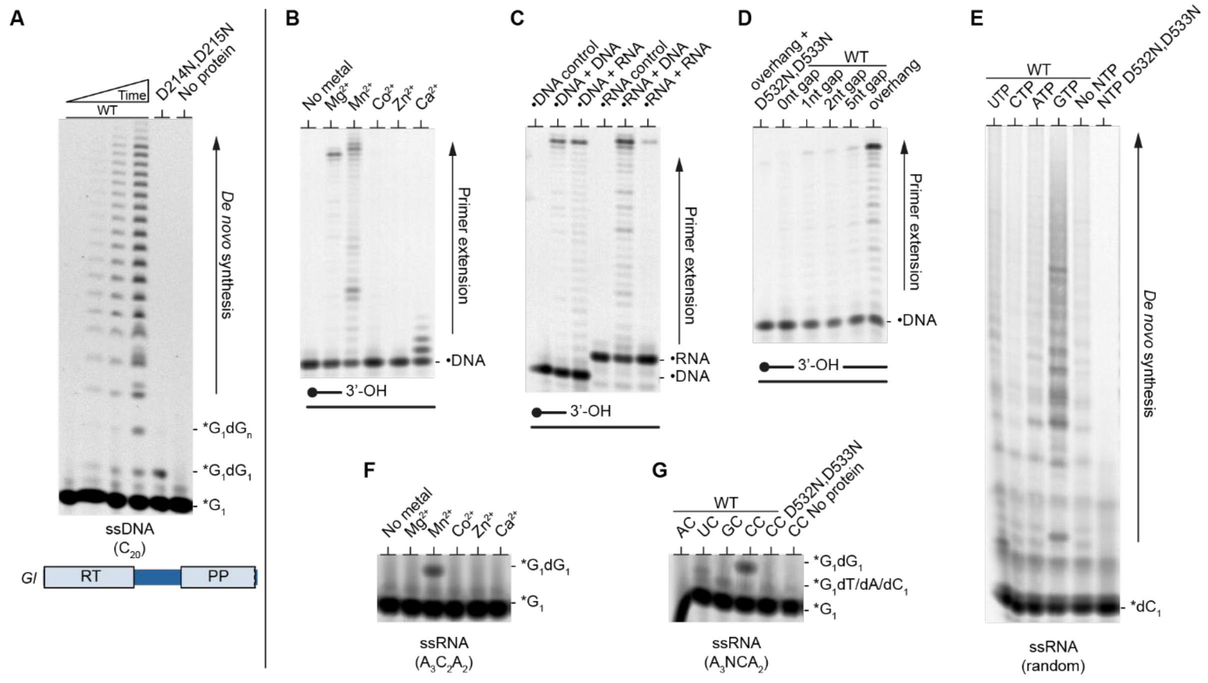

Figure S2: Enzymatic properties of *Gl*CART-CAPP and *Mm*Cas6-CART-Cas1

(A) Time course the primase activity of *Gl*CART-CAPP RT-PP protein domain fusion. 1 μM protein was incubated with 1 μM DNA (oMZ13), γ-phosphate FAM-labelled GTP, dGTP and Mn^{2+}. Time course: 0, 2, 5 and 30 min. No protein control and RT catalytic mutant D214N, D215N of the RT domain were incubated for 30 min. **(B)** Divalent metal cation preference of RT domain of *Mm*Cas6-CART-Cas1 for the primer extension activities, showing Mg^{2+} and Mn^{2+} promote extension of DNA substrate (oPK404, oPK405) with dNTPs. **(C)** The *Mm*Cas6-CART-Cas1 primer extension activities of DNA and RNA primers (oPK405 and oPK407) on DNA and RNA templates (oPK404 and oPK406) with dNTPs in presence of Mg^{2+}. **(D)** *Mm*Cas6-CART-Cas1 shows poor strand-displacement synthesis activity. The extension of DNA primer (oNB2) on DNA template (oNB1) is inefficient as if different strands (oNB8-11) are annealed downstream of the primer in presence of Mg^{2+} and dNTPs. **(E)** The *de novo* DNA

synthesis of *Mm*Cas6-CART-Cas1 is not majorly stimulated by NTPs. Priming activity of *Mm*Cas6-RT-Cas1 was tested in presence or absence of UTP, CTP, ATP or GTP, Mn^{2+}, dATP, dGTP, dTTP, FAM-labelled dCTP and random sequence RNA template (oKZ151). **(F)** The metal preference of *Mm*Cas6-RT-Cas1 in the primase assay showing only Mn^{2+} promotes *de novo* DNA synthesis on RNA template (oMZ12) with dGTP and γ-phosphate FAM-labelled GTP. **(G)** Sequence preference of *Mm*Cas6-RT-Cas1 for the initiation of *de novo* synthesis. RNA substrate heptamers with the initiation sequence indicated (AC, oMZ18; CC, oMZ12; UC, oMZ19; GC, oMZ20 – initiating template base is underlined) were tested in presence of Mn^{2+}, γ-phosphate FAM-labelled GTP and respective second complementary nucleotide. D532N, D533N indicates catalytic mutation in RT domain of *Mm*Cas6-CART-Cas1. Products of all reactions were resolved on TBE-urea-PAGE gel (Panel **A** – **G**). •DNA, FAM-labelled DNA primer; •RNA, FAM-labelled RNA primer; *dC, FAM-labelled dCTP; *G, γ-phosphate FAM-labelled GTP. The template sequence: 5' → 3'.

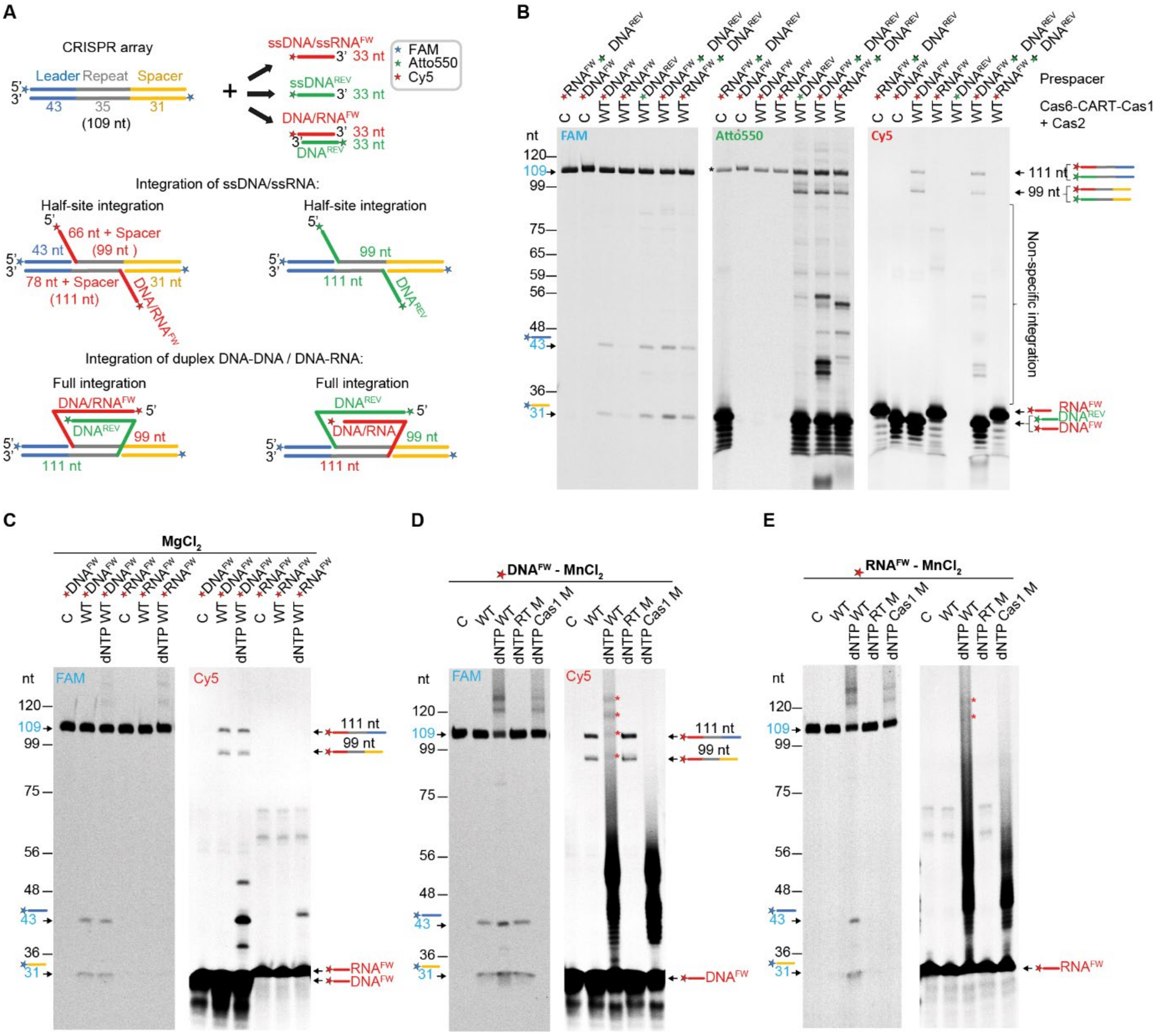

Figure S3: *Mm*Cas6-CART-Cas1 – Cas2 is inefficient in direct RNA integration into CRISPR array

(A) Graphical representation of *Mm*CRISPR array and prespacers used in the prespacer integration assay (top) and putative *Mm*Cas6-CART-Cas1 – Cas2 integration products (middle and bottom). The length of products is indicated. **(B)** *Mm*Cas6-CART-Cas1 integrates DNA strands, but not RNA strands, into the *Mm*CRISPR array in absence of dNTPs. 1 µM *Mm*Cas6-CART-Cas1 wild-type (WT) with 2 µM *Mm*Cas2 were incubated with 200 nM prespacer and 25 nM *Mm*CRISPR array A in presence of 10 mM Mn^{2+} for 1 hour at 37 °C. **(C)** *Mm*Cas6-CART-Cas1

extends DNA and RNA prespacers in presence of dNTPs and Mg^{2+} and efficiently integrates DNA spacers into *Mm*CRISPR array. Reaction conditions were used as above in the presence of Mg^{2+} and presence or absence of 100 μM dNTPs. **(D)** *Mm*Cas6-CART-Cas1 efficiently extends and integrates DNA prespacer into CRISPR array in presence of Mn^{2+} and dNTPs. Reaction conditions were used as above (Panel **B**) in presence or absence of 100 μM dNTPs. **(E)** *Mm*Cas6-CART-Cas1 efficiently integrates DNA-extended RNA prespacer into CRISPR array in presence of Mn^{2+} and dNTPs. The reaction conditions were used as above (Panel **B**) in presence or absence of 100 μM dNTPs. Nt, nucleotides. *Mm*Cas6-CART-Cas1 D532N, D533N (RT catalytic mutant, RT M), *Mm*Cas6-CART-Cas1 H855A, E870A (Cas1 catalytic mutant, Cas1 M). Arrows on both sides of the gel indicate CRISPR integration products. Red stars indicate integration of extended prespacers. C, control without protein; nt, nucleotides. The length markers are indicated on the left of the gels. Results are representative of three independent experiments (**B** – **E**).

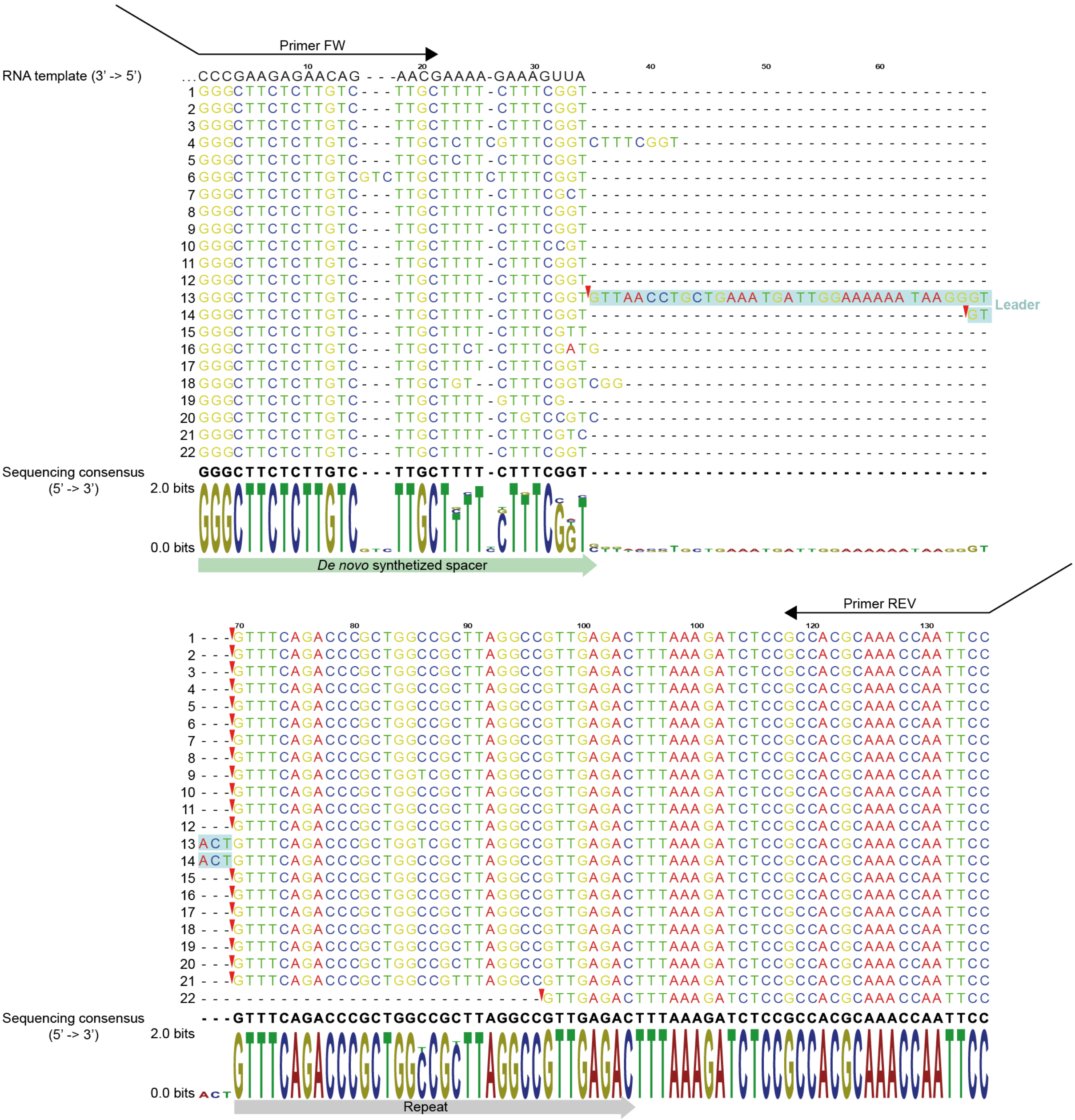

Figure S4: Full list of sequenced spacers integrated into *Mm*CRISPR array from *in vitro* primed prespacer integration assay.

Black arrows indicate primers (Forward, FW; Reverse, REV). Red triangle indicates position of prespacer integration into the *Mm*CRISPR array. Sequence logo – the size of the bases indicates their occurrence. Numbers on the left indicates sample number. For simplicity, 6 out of 22 sequenced samples were omitted in Figure **4E** as three of

the samples (sample 13, 14, 22) showed integration of the spacer out of the leader-repeat junction, one (sample 6) had a mutation in the primer, one (sample 16) contained adenine base in the spacer which was probably artificially integrated during PCR amplification and one (sample 4) had a very long spacer. Sequences with blue background are sequences of the leader.

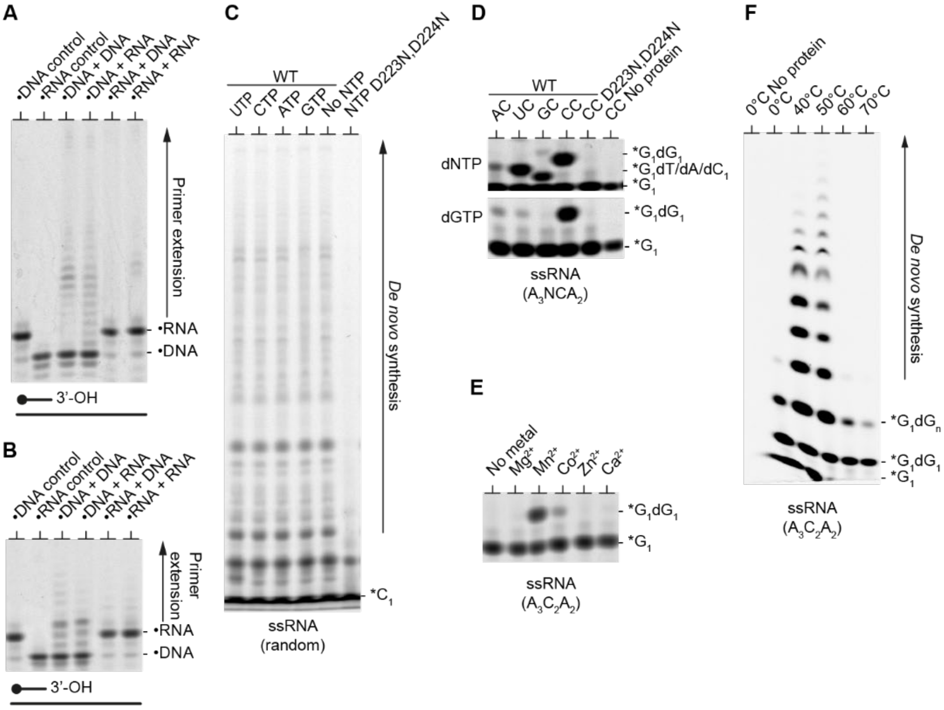

Figure S5: Enzymatic properties of *Gs*RT

(A, B) Primer extension activity of *Gs*RT with DNA and RNA primers (oPK405 and oPK407) on DNA and RNA templates (oPK404 and oPK406) in presence of Mn^{2+} with dNTPs (Panel **A**) or NTPs (Panel **B**). **(C)** *Gs*RT *de novo* DNA synthesis is not stimulated by NTPs. The addition of either UTP, CTP, ATP or GTP did not increase the yield of primers, compared to reaction without NTPs (none). The random RNA sequence (oKZ151) was used as non-discriminatory template together with dATP, dTTP, dGTP, FAM-dCTP in presence of Mn^{2+}. **(D)** Sequence preference of *Gs*RT for the initiation of *de novo* synthesis. RNA substrate heptamers with the initiation sequence indicated (A<u>C</u>, oMZ18; C<u>C</u>, oMZ12; U<u>C</u>, oMZ19; G<u>C</u>, oMZ20 – initiating template base is underlined) were tested with γ-phosphate FAM-labelled GTP and respective second complementary nucleotide (top) in presence of Mn^{2+}. Similar reaction with just dGTP and γ-phosphate FAM-labelled GTP exemplifies base-pairing specificity is essential (bottom). D223N, D224N indicates catalytic mutant of *Gs*RT.

(E) The metal preference of *Gs*RT in primase assays showing Mn^{2+} and Co^{2+} promote *de novo* DNA synthesis on RNA template (oMZ12) with dGTP and γ-phosphate FAM-labelled GTP. **(F)** The optimal temperature of *Gs*RT in primase assays, showing *de novo* DNA synthesis on RNA template (oMZ12), with dGTP and γ-phosphate FAM-labelled GTP, in temperature range 0-70 °C in presence of Mn^{2+}. With the increasing temperature, a prominent shift in size of the products indicates a non-specific extension beyond the template length. Products of all reactions were resolved on TBE-urea-PAGE gel (Panel **A** – **F**). •DNA, FAM-labelled DNA primer; •RNA, FAM-labelled RNA primer; *dC, FAM-labelled dCTP; *G, γ-phosphate FAM-labelled GTP. The template sequence: 5' → 3'.

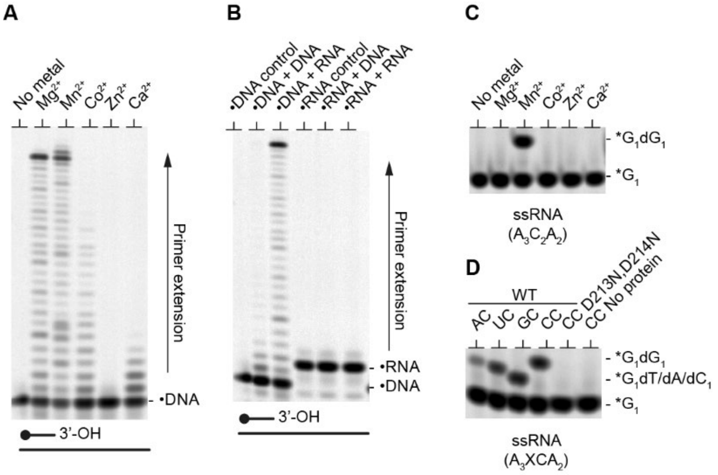

Figure S6: Enzymatic properties of *Sc*Ty3 RT domain

(A) The divalent metal cation preference of *Sc*TY3 RT domain for the primer extension activity showing Mg^{2+} and Mn^{2+} promote efficient extension of DNA-primer:RNA-template substrate (oPK405 + oPK406) with dNTPs. **(B)** Primer extension activity of RT domain of *Sc*Ty3 with DNA and RNA primers (oPK405 and oPK407) on DNA and RNA templates (oPK404 and oPK406) with dNTPs in presence of Mg^{2+}. **(C)** The metal preference of *Sc*Ty3 RT domain in primase assays showing that Mn^{2+} promotes *de novo* DNA synthesis on RNA template (oMZ12) with dGTP and γ-phosphate FAM-labelled GTP. **(D)** Sequence preference for the initiation of *de novo* synthesis by *Sc*TY3 RT domain. RNA substrate heptamers with the initiation sequence indicated (AC, oMZ18; CC, oMZ12; UC, oMZ19; GC, oMZ20 – initiating template base is underlined) were tested with γ-phosphate FAM-labelled GTP and their respective complementary second nucleotide. All reaction products were resolved on TBE-urea-PAGE gel (Panel **A** – **D**). •DNA, FAM-labelled DNA primer; •RNA, FAM-labelled RNA primer; *G, γ-phosphate FAM-labelled GTP. The template sequence: 5' → 3'.

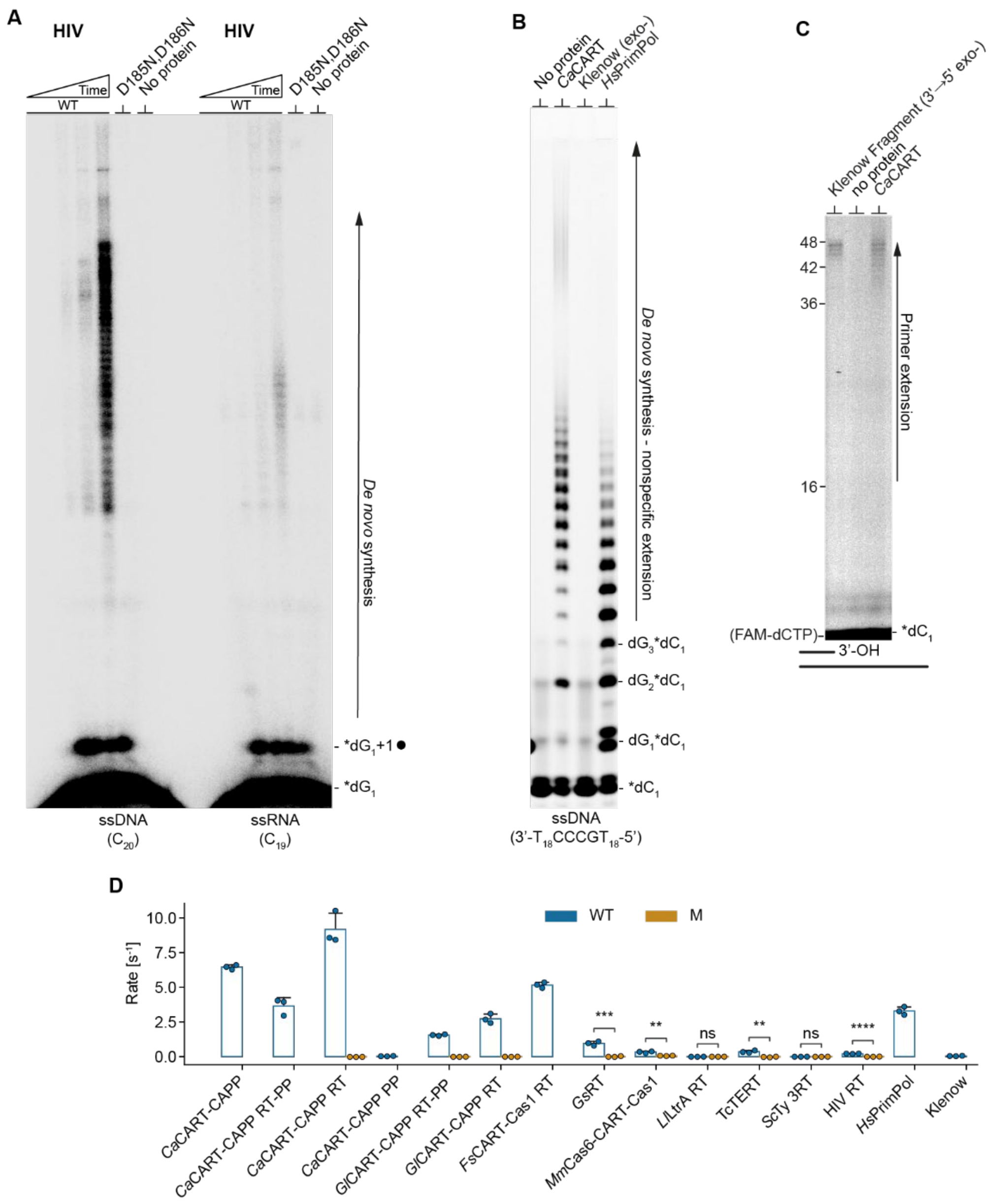

Figure S7: Primase activity of HIV and a comparison of primase activities of RTs with *Hs*PrimPol

(A) HIV RT is able to prime on ssDNA and ssRNA templates. Time course of HIV RT (p66 - p51 heterodimer) primase activity, with 1 µM protein incubated with α-32P-labelled dGTP and dGTP, with 1 µM ssDNA (oMZ13) or ssRNA (oMZ27). The gel was developed for 1 hour on BAS-MS imaging plate (Fujifilm) and scanned on IP stage of FLA 5100 (Fujifilm). D185N, D186N represents catalytic mutant of both subunits of

HIV RT. Black spot indicates position of synthesized dinucleotides. **(B)** Klenow (exo-) has no primase activity. Gel-based primase assay with 1 μM protein (*Ca*CART-CAPP RT, *Hs*PrimPol, Klenow (exo-)), Mn^{2+}, dGTP, FAM-dCTP and 1 μM ssDNA template (oKZ364) incubated for 30 min. **(C)** Klenow (exo-) incorporates FAM-labelled dCTP during primer extension. Gel-based polymerase assay comparing the incorporation of FAM-labelled dCTP in presence of 50 nM protein (*Ca*CART-CAPP RT, Klenow (exo-)), Mn^{2+}, dGTP/dATP/dTTP and 50 nM DNA primer (oPK405-no FAM) annealed with DNA template (oPK404), incubated for 30 min. **(D)** The comparison of RTs priming activities. The intercalating fluorescent dye-based primase assay with 250 nM protein on 1 μM ssDNA (oKZ148, $C_{20}T_{20}$) and statistical analysis with two-tailed independent t-test. **, p-value < 0.01; ***, p-value < 0.001; **/*, p-value < 0.0001; ns, not significant.

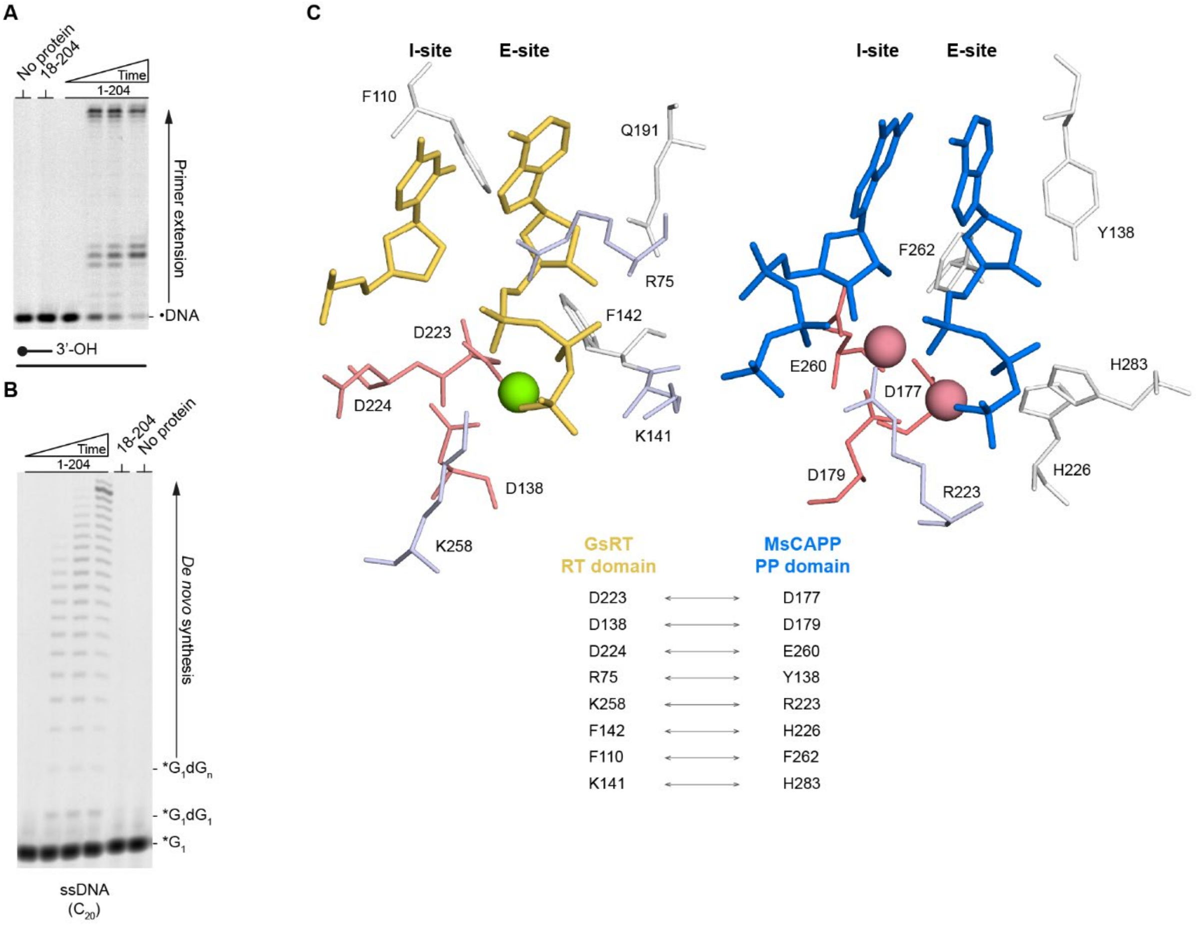

Figure S8: N-terminal β-hairpin of *Ca*CART-CAPP RT is crucial for enzyme activity

(A) Polymerase activity of *Ca*CART-CAPP RT is abolished if the N-terminal β-hairpin is deleted. 50 nM protein was incubated with 50 nM DNA substrate (oPK404 + oPK405) in presence of dNTPs and Mg^{2+}. Time course: 0, 5, 10 and 30 min. •DNA, FAM-labelled DNA primer. **(B)** N-terminal β-hairpin is essential for *Ca*CART-CAPP RT domain priming activity. 1 μM protein was incubated with 1 μM DNA substrate (oMZ13) in presence of dGTP, γ-phosphate FAM-labelled GTP and Mn^{2+}. Time course: 0, 2, 10 and 30 min. Products of polymerase and primase assays were resolved on TBE-Urea-PAGE gels (Panel **A** – **B**). No protein control, and truncated RT aa 18 - 204 were incubated for 30 min. Active full-length RT domain is aa 1 - 204; *G, γ-phosphate FAM-

labelled GTP. **(C)** A close-up view of the catalytic sites of *Gs*RT (6ar1) and *Ms*CAPP (7p9j). The nucleotides are coloured by the source structure colour *Gs*RT (yellow), *Ms*CAPP (blue) with annotated positions: initiating (I-site), extending (E-site). The metals are coloured by element Mg^{2+} (green) and Mn^{2+} (pink). The active site residues are coloured by hydrophobicity scale: polar cation (light blue), polar anion (pink), aliphatic (white).
