# Supplementary material for: Reverse transcriptases prime DNA synthesis
Source: Nucleic Acids Res. 2023 Jun 6;51(14):7125–42. doi: 10.1093/nar/gkad478 (PMC10415136; doi:10.1093/nar/gkad478)
Supplement: gkad478_Supplemental_Files [file gkad478_supplemental_files.zip › Supplementary table legends.docx]

**Supplementary Table legends**

**Supplementary Table 1**. A list of plasmids used in this study

**Supplementary Table 2**. Proteins used in this study, including their expression and purification conditions

**Supplementary Table 3**. Sequences of primers and DNA constructs used in this study

**Supplementary Table 4**. Polymerase assays: a list of detailed reaction conditions of each experiment

**Supplementary Table 5**. Primase assays: a list of detailed reaction conditions of each experiment

**Supplementary Table 6**. Intercalating fluorescent dye-based primase assay: a list of detailed reaction conditions for each experiment

**Supplementary Table 7**. Results obtained following searches of the PDB, using the Dali structure threading server, for structural homologues of the RT domain from *Ca*CART-CAPP
